# Marked Global DNA Hypomethylation Is Associated with Constitutive PD-L1 Expression in Melanoma

**DOI:** 10.1016/j.isci.2018.05.021

**Published:** 2018-06-28

**Authors:** Aniruddha Chatterjee, Euan J. Rodger, Antonio Ahn, Peter A. Stockwell, Matthew Parry, Jyoti Motwani, Stuart J. Gallagher, Elena Shklovskaya, Jessamy Tiffen, Michael R. Eccles, Peter Hersey

**Affiliations:** 1 Department of Pathology, Dunedin School of Medicine, University of Otago, 270 Great King Street, Dunedin 9054, New Zealand; 2 Maurice Wilkins Centre for Molecular Biodiscovery, Level 2, 3A Symonds Street, Auckland, New Zealand; 3 Department of Mathematics & Statistics, University of Otago, 710 Cumberland Street, Dunedin 9054, New Zealand; 4 Melanoma Immunology and Oncology Group, The Centenary Institute, University of Sydney, Royal Prince Alfred Hospital, Missenden Road, Camperdown, NSW 2050, Australia

**Keywords:** Genetics, Genomics, Cancer, Transcriptomics

## Abstract

Constitutive expression of the immune checkpoint, PD-L1, inhibits anti-tumor immune responses in cancer, although the factors involved in PD-L1 regulation are poorly understood. Here we show that loss of global DNA methylation, particularly in intergenic regions and repeat elements, is associated with constitutive (PD-L1_CON_), versus inducible (PD-L1_IND_), PD-L1 expression in melanoma cell lines. We further show this is accompanied by transcriptomic up-regulation. *De novo* epigenetic regulators (e.g., *DNMT3A*) are strongly correlated with PD-L1 expression and methylome status. Accordingly, decitabine-mediated inhibition of global methylation in melanoma cells leads to increased PD-L1 expression. Moreover, viral mimicry and immune response genes are highly expressed in lymphocyte-negative plus PD-L1-positive melanomas, versus PD-L1-negative melanomas in The Cancer Genome Atlas (TCGA). In summary, using integrated genomic analysis we identified that global DNA methylation influences PD-L1 expression in melanoma, and hence melanoma's ability to evade anti-tumor immune responses. These results have implications for combining epigenetic therapy with immunotherapy.

## Highlights

• Global DNA hypomethylation in melanoma promotes constitutive PD-L1 expression
• Transcriptomic up-regulation accompanies constitutive PD-L1 expression in melanoma
• Constitutive PD-L1 is associated with reduced *DNMT3A* expression and viral mimicry
• DNA methylation inhibitor treatment of melanoma cells increases PD-L1 expression

## Graphical Abstract

**Figure:**
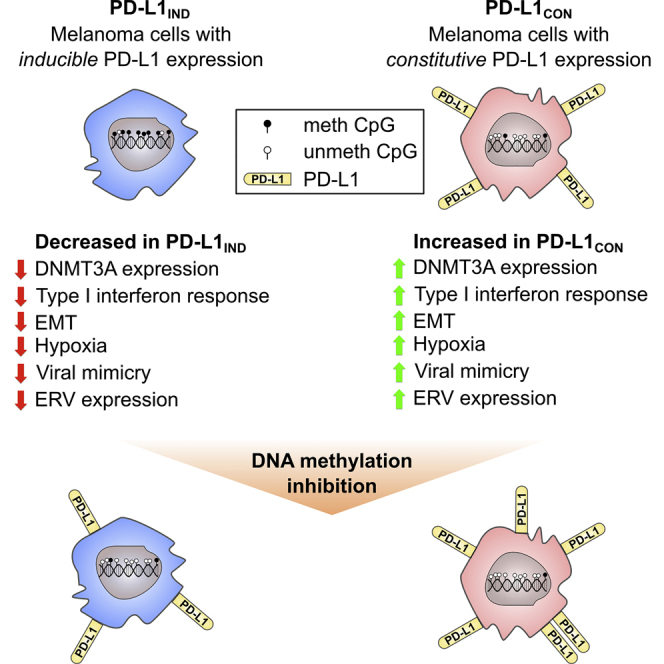

## Introduction

Immunotherapy with monoclonal antibodies (mAbs) that block PD-1/PD-L1 interactions on immune cells has shown remarkable success in the treatment of melanoma (Hersey and Gowrishankar, 2015, Hodi et al., 2016, Ribas et al., 2016, Ugurel et al., 2016) and other malignancies (Gandini et al., 2016, Ansell et al., 2015). PD-1 (*CD279*) is an inhibitory molecule that inhibits T-cell receptor (TCR) signaling on T cells by increasing the threshold necessary for their activation and effector function. As such, it is often expressed on activated CD4 and CD8 T cells, but other immune cells (e.g., NK and B cells and monocytes) may also express this molecule. PD-1 engages primarily with its ligands PD-L1 (B7-H1, *CD274*) and PD-L2 (B7-DC, *CD273*) (Greenwald et al., 2005, Yao et al., 2013), which are widely expressed not only on immunocompetent cells but also in non-lymphoid organs (Dong et al., 2004, Greenwald et al., 2005).

Melanoma cells commonly express PD-L1 as an adaptive response to T cell recognition (Gowrishankar et al., 2015, Ribas, 2015). This results in activation of the inhibitory PD-1 receptor on T cells that infiltrate the tumor microenvironment, resulting in inhibition of their activity (Schildberg et al., 2016) and allowing melanoma cells to evade the host immune system. Up-regulation of PD-L1 on melanoma cells is believed to result from interferon gamma (IFN-γ) release by T cells that interact with the tumor. IFN-γ then signals through the type II IFN receptor by activating the JAK/STAT pathway. STAT then interacts in the nucleus with IFN-γ activation sites (GAS) in the promoters of IFN-stimulated genes (Platanias, 2005).

A number of studies have examined whether PD-L1 may be a biomarker to help select responders to anti-PD-1 inhibitors, but as a single marker it has been of limited value mainly because of its variable expression (Madore et al., 2014) and the detection of responses in patients with PD-L1-negative tumors (Daud et al., 2016, Festino et al., 2016). We (Madore et al., 2014) and others (Smyth et al., 2016, Taube et al., 2012, Topalian et al., 2016) have shown that patients can be subdivided into at least four groups depending on the expression of PD-L1 and T lymphocyte infiltration (TIL). In a large proportion of patients (approximately 30%) PD-L1 expression is associated with infiltration of the tumor by TILs (TIL+/PD-L1+). In 20% there are TILs but no PD-L1 expression (TIL+/PD-L1−), and in 40% there are neither TILs nor PD-L1 expression (TIL−/PD-L1−) (Ribas, 2015, Topalian et al., 2016). The remaining melanomas exhibit diffuse expression of PD-L1 without the presence of TILs (TIL−/PD-L1+), which is referred to as constitutive PD-L1 expression. Previous authors have speculated that constitutive expression is due to oncogene-driven expression (Pardoll, 2012), but we (Gowrishankar et al., 2015) and others have excluded a number of potential oncogenic pathways that have been implicated in other cancers (Spranger and Gajewski, 2016).

To better understand the basis for constitutive expression of PD-L1 we have examined whether epigenetic mechanisms play a potential role in the regulation of PD-L1 expression. Previous studies in non-small cell lung cancer cell lines have shown up-regulation of PD-L1 after treatment with the demethylating agent azacytidine (Wrangle et al., 2013). Similar findings were reported in studies on breast, colon, and ovarian carcinoma lines (Li et al., 2014). Additional evidence that DNA methylation may constitute an additional regulatory mechanism came from Madore et al. (2016) who found that low or absent PD-L1 expression in 52 patients with melanoma in The Cancer Genome Atlas (TCGA) was associated with high levels of DNA methylation, as assessed using Illumina 450K arrays. In view of these findings, we have explored whether epigenetic mechanisms associated with DNA methylation could underlie the constitutive expression of PD-L1 on melanoma, by either silencing repressive factors or by activation of pathways that normally regulate PD-L1 expression.

## Results

### Characterization of Inducible and Constitutive Patterns of Expression of PD-L1 (PD-L1_IND_ and PD-L1_CON_) in Melanoma Cell Lines

**Figure 1:**
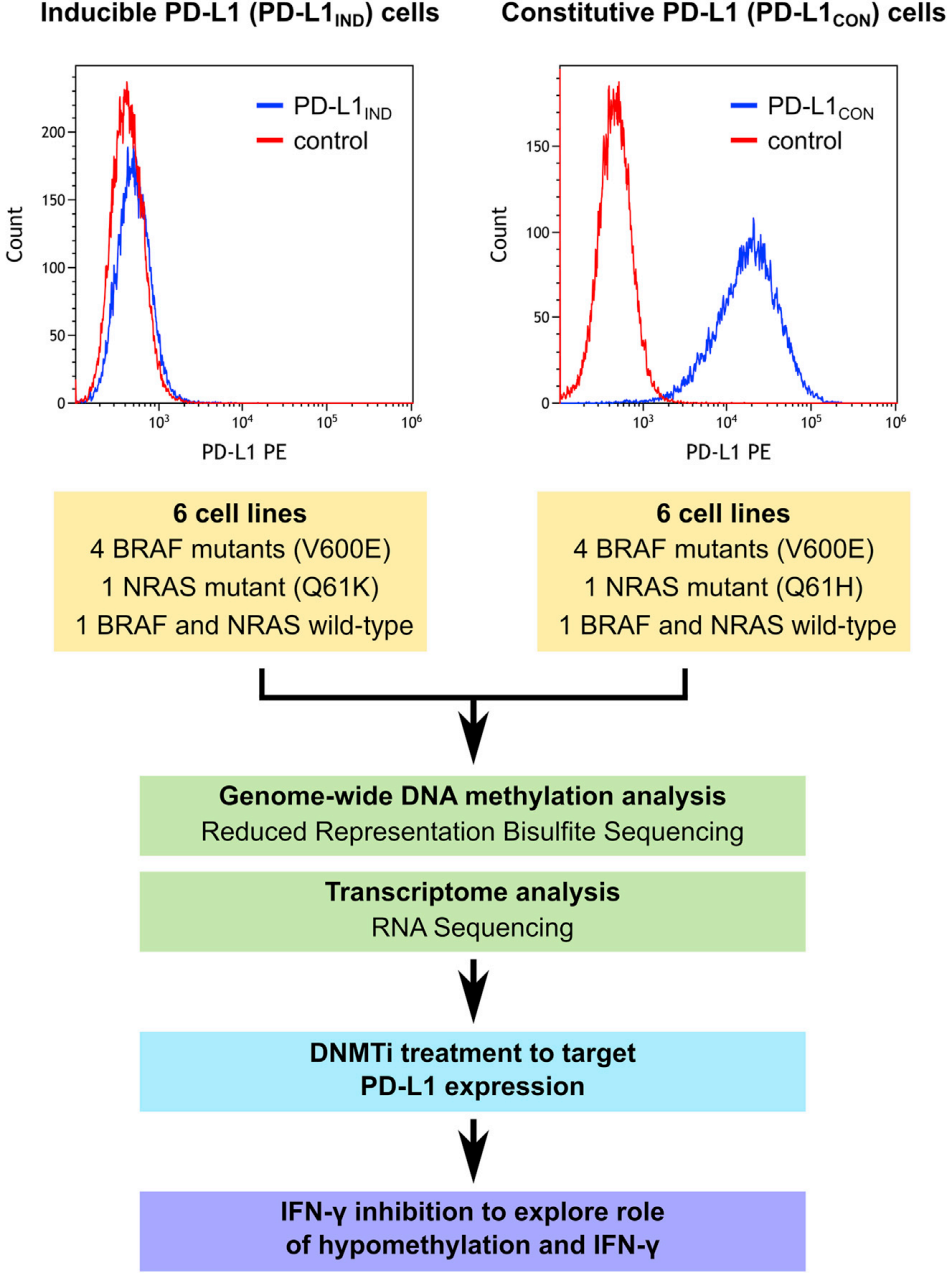
Summary of Experimental Design and the Analysis Pipeline for PD-L1_IND_ and PD-L1_CON_ Cell Lines to Identify Epigenetic Regulation of PD-L1 in Melanoma For a Figure360 author presentation of Figure 1, see http//dx.doi:10.1016/j.isci.2018.05.021#mmc3. The upper panel shows representative FACS figures from PD-L1_CON_ and PD-L1_IND_ cells. See also Figures S1–S3, and Table S1. Figure360: An Author Presentation of Figure 1

To characterize the expression patterns of PD-L1 in melanoma, we investigated cell surface PD-L1 expression in melanoma cell lines and selected six cell lines that constitutively expressed PD-L1 (PD-L1 positive, referred to as PD-L1_CON_) and six cell lines that expressed PD-L1 only upon induction after treatment with IFN-γ (PD-L1 negative, referred to as PD-L1_IND_) (Figure 1). The percentage of PD-L1-positive cells in PD-L1_CON_ cell lines ranged from 41.6% to 99.07% (median = 93.57%), whereas the proportion of PD-L1-positive cells in PD-L1_IND_ was confirmed to be very low (0.82%–6.79% [median = 1.7%, Figure 1, details in Table S1 and Figures S1 and S2]). Some PD-L1_CON_ cell lines constitutively produced significant amounts of IFN-γ (Gallagher et al., 2014), and therefore we considered the possibility of a role for IFN-γ feedback in maintaining the constitutive PD-L1 expression on these cells. However, blockade of interferon type I or type II signaling did not affect constitutive PD-L1 expression in two PD-L1_CON_ cell lines (Figure S3). Furthermore, the presence of common oncogenic driver mutations was similar between each group of cell lines; each group contained four cell lines harboring a *BRAFV600E* mutation, one with a *NRASQ61* mutation and one wild-type for *NRAS* and *BRAF* (Table S1). These data suggest that factors other than IFN-γ, or oncogenic signaling, are involved in regulating PD-L1 expression.

### Whole-Genome-Scale DNA Methylation Identifies Extensive Global Hypomethylation in Constitutive PD-L1 Cell Lines (PD-L1_CON_)

**Figure 2:**
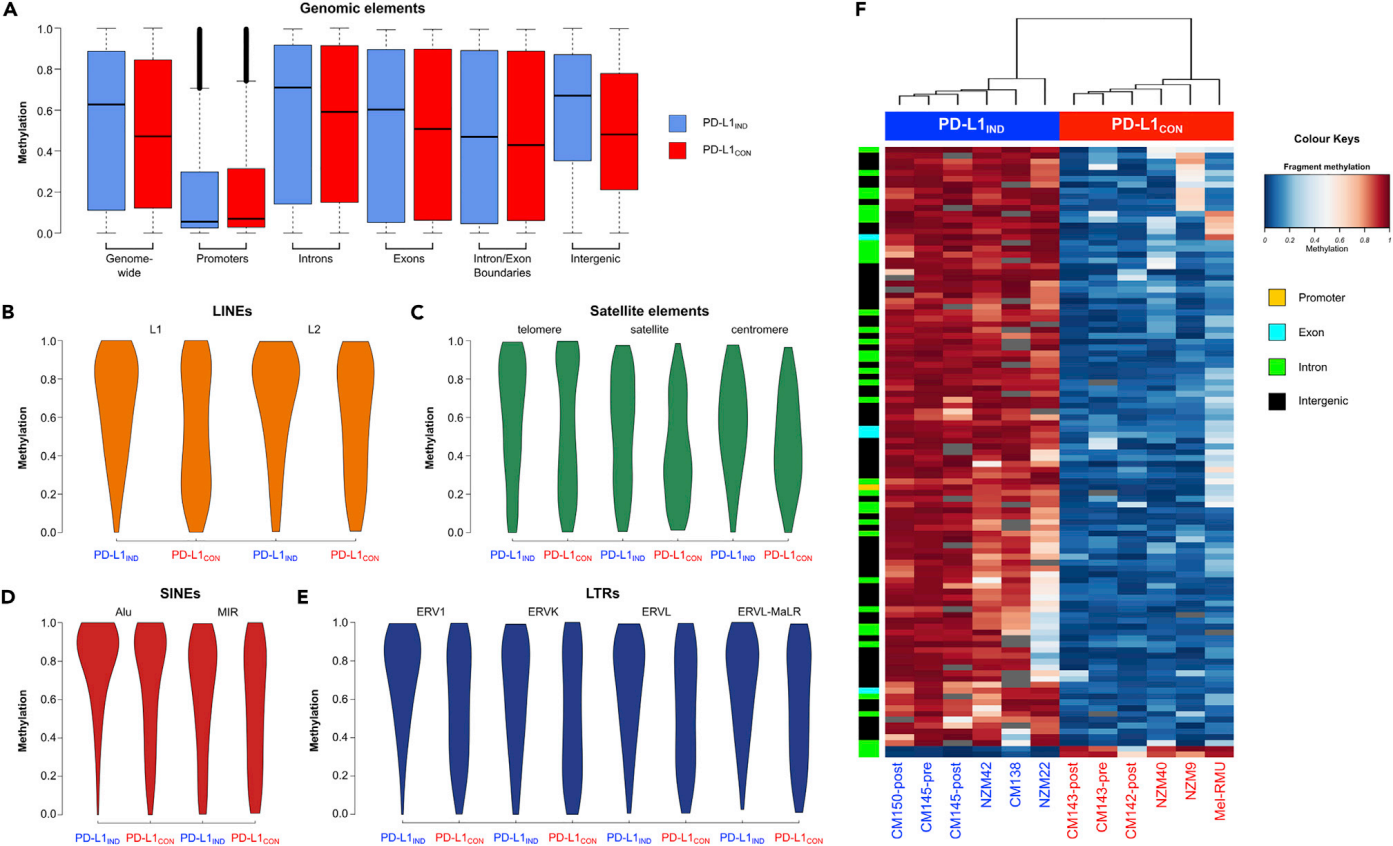
Whole-Genome-Scale and Element-Wise Methylation Profiles in PD-L1_IND_ and PD-L1_CON_ Cell Lines (A) Boxplots showing genome-wide and genomic element RRBS methylation profiles for PD-L1_IND_ (blue) and PD-L1_CON_ (red) cell lines; black bars indicate the median methylation. (B–E) Equal-area violin plots of PD-L1_CON_ and PD-L1_IND_ DNA methylation levels for different classes of repeat elements. (B) LINE elements (L1 and L2), (C) Satellite elements (satellite, telomeric, and centromeric repeats), (D) SINE elements (Alu and MIR), and (E) LTRs (ERV1, ERVK, ERVL, and ERVL-MaLR). In all cases the y axis represents the methylation level on a 0–1 scale. Annotations for repeat elements were downloaded from the UCSC repeat masker database. (F) Methylation levels for the 105 differentially methylated fragments (DMFs) showing >70% methylation difference between the PD-L1_IND_ and PD-L1_CON_ cell lines (blue = unmethylated, red = fully methylated). See also Figures S4–S9 and Tables S2–S4. The methylation data are available at Database: NCBI GEO, accession number GSE107622.

We generated genome-scale DNA methylation maps by performing reduced representation bisulfite sequencing (RRBS) (Chatterjee et al., 2017b, Meissner et al., 2008) on the PD-L1_IND_ and PD-L1_CON_ lines. In total, we obtained 535 million sequence reads for the 12 cell lines, allowing the investigation of 290,385 MspI fragments consisting of 1.66 million reads at a high coverage (Chatterjee et al., 2017a) (Table S2). The striking finding from this analysis was that global genomic methylation levels in the PD-L1_CON_ cell lines were much lower than those in PD-L1_IND_ cell lines (median methylation = 0.47 and 0.63, respectively, Wilcoxon rank test p value <2.2 × 10^−16^, Figure 2 and Table S3). The hypomethylation of PD-L1_CON_ cell lines was particularly pronounced in intergenic regions and in gene introns (Figure 2A). Intergenic regions showed a 19% median methylation reduction in PD-L1_CON_, whereas for introns the median loss of methylation was 12%. Gene promoters (defined as −5 kb to +1 kb) were hypomethylated in both groups, and exon regions showed similar levels of methylation in both groups (Figure 2A and Table S3).

PD-L1_CON_ cells showed hypomethylation in every class of repeat element analyzed (Figures 2B–2E and Table S4). Although hypomethylation was consistent in all repeat regions, the degree of methylation loss varied between subfamilies of repeats and individual elements. The LTR family showed the highest degree of hypomethylation in PD-L1_CON_ compared with PD-L1_IND_ cells (Figure 2E). For LTRs, the loss of median methylation ranged from 13% to 19%, with ERV1 showing the most significant hypomethylation. For LINE elements, the evolutionarily younger L1 showed a higher degree of hypomethylation (median methylation = 0.72 and 0.53 in PD-L1_IND_ and PD-L1_CON_, respectively, Figure 2B) than the evolutionarily older L2 element (median methylation 0.75 and 0.64 in PD-L1_IND_ and PD-L1_CON_ lines, respectively).

Next, we identified 1,180 differentially methylated fragments (DMFs, F test at 5% false discovery rate (FDR) with 25% mean methylation difference for a fragment) that were mostly hypomethylated (96.4% of the DMFs) in PD-L1_CON_ cell lines, consistent with the global patterns. There was a large difference in methylation levels (>50%) in three-quarters of the DMFs (Figures S4 and S5), and we identified 105 regions that showed >75% methylation differences between the PD-L1_IND_ and PD-L1_CON_ groups (Figure 2F). The strikingly divergent methylation pattern between the inducible and constitutive lines suggests there may be a common methylation-associated regulatory mechanism between the two groups.

To compare the RRBS methylation profiles observed in PD-L1_CON_ and PD-L1_IND_ cell lines with that of melanoma tumors, we analyzed 450K DNA methylation data from the TCGA-SKCM cohort. We specifically analyzed tumors that were TIL− to reduce the impact of immune cell signaling on tumor PD-L1 expression, and we divided these tumors into group 1 (PD-L1−, n = 180) and group 2 (PD-L1+, n = 54). We considered these two groups as being the most representative of our analyzed inducible (group 1) and constitutive cell lines (group 2, Figures S6 and S7). We could not detect a significant global methylation difference between group 1 and group 2 melanomas (Figure S8), which we surmise is because of the promoter-biased design of the 450K probes and which suggests that RRBS has better discrimination power than the 450K platform to detect methylation differences in intergenic regions, introns, and repeat elements of melanomas. In addition, we specifically examined the five CpG island-associated probes in the promoter and 5′ untranslated region (5′UTR) of the gene for PD-L1 (*CD274*) in TCGA data. CpG probes cg02823866 and cg14305799 were located within 200 bp of the transcription start site (TSS) in the *CD274* promoter, whereas cg15837913 was located within 1,500 bp from the TSS and cg13474877 together with cg19724470 were within the *CD274* 5′UTR. The two CpGs in the *CD274* promoter were essentially unmethylated (<5% mean methylation) in both melanoma groups, whereas the two CpGs in the 5′UTR showed a loss of methylation (13% for cg15837913 and 16% for cg19724470) in group 2 (representative of constitutive melanoma) compared with group 1 (inducible melanoma, Figures S9A–S9E). These results are consistent with our RRBS data. We also analyzed the correlation of methylation in these probes with mRNA expression in the same patient groups and found the 5′UTR-associated cg19724470 methylation was significantly negatively correlated with PD-L1 expression (r = 0.49, p value = 1.44 × 10^−15^, Figures S9F–S9J). These observations allude to the possibility that epigenetic modification of a distal enhancer or other distant elements might be involved in the regulation of *CD274* gene expression and identifying these elements would consequently be required for a full understanding of the overall regulatory processes controlling *CD274* gene expression. However, the impact of this difference in the methylation of a single CpG in the 5′UTR on the overall expression of *CD274* presently remains unclear. Nevertheless, we found that, overall, there was an insignificant difference in methylation of the *CD274* core promoter between the two groups.

### Transcriptomic Features Reveal Distinct Differences in Expression Patterns between Constitutive and Inducible PD-L1 Lines

**Figure 3:**
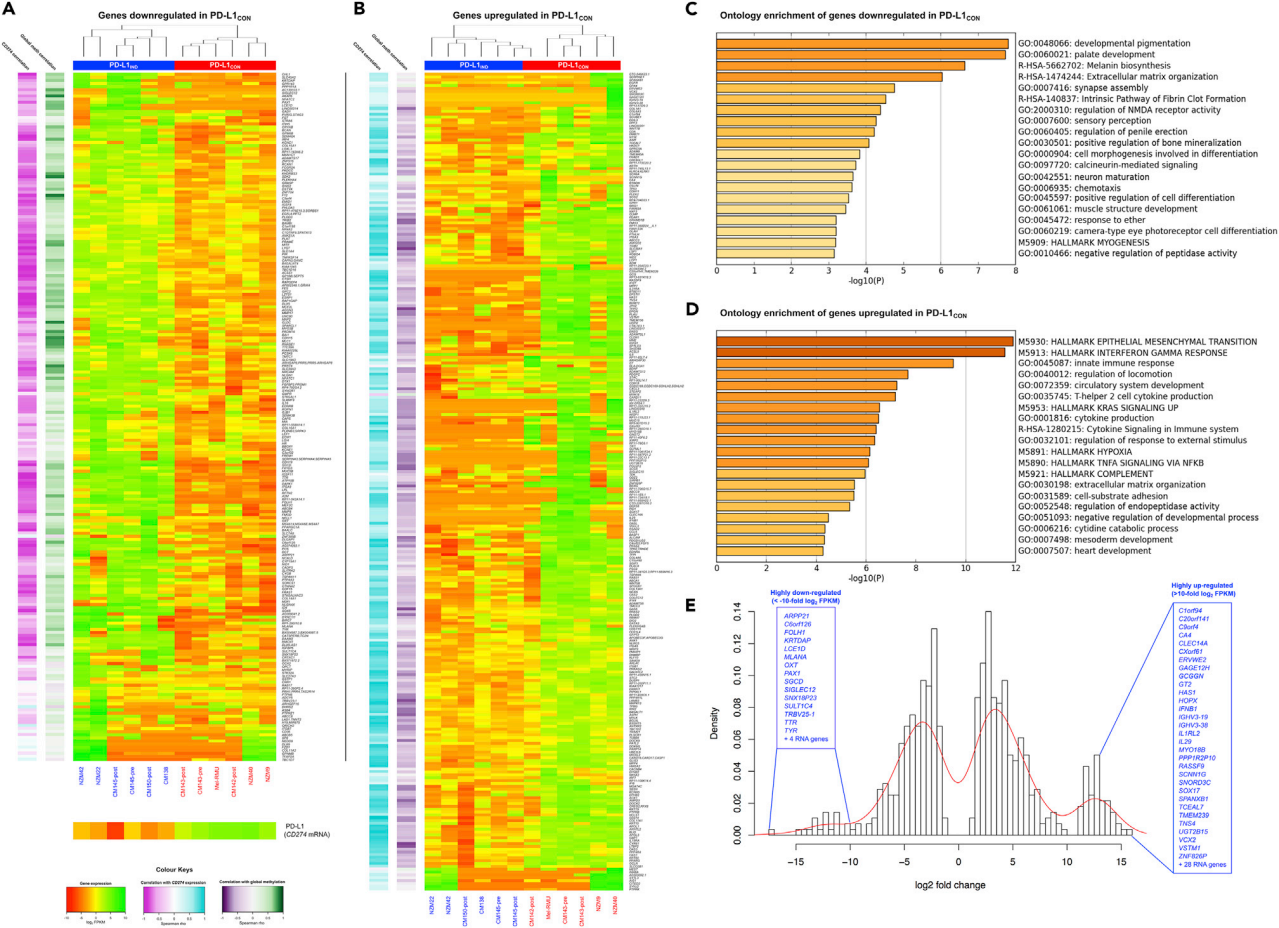
Differential Expression Patterns in PD-L1_IND_ and PD-L1_CON_ Cell Lines (A) Mean-centered heatmap of the expression level (log_2_ FPKMs) of 222 significantly down-regulated genes in PD-L1_CON_. (B) Mean-centered heatmap of the expression level (log_2_ FPKMs) of 286 significantly up-regulated genes in PD-L1_CON_. The correlations of these genes with *CD274* (PD-L1) expression and global methylation status in the analyzed cell lines are shown in the colored sidebars (left) in both figures. (C) Enriched gene ontology terms relative to the 222 genes down-regulated in PD-L1_CON_ cell lines. (D) Enriched gene ontology terms relative to the 286 genes up-regulated in PD-L1_CON_ cell lines. In figure (C) and (D), the x axis represents –log_10_ of the p value. (E) Density histogram of the log_2_ fold changes for the significantly up-regulated (n = 286, right side of the histogram) and down-regulated (n = 222, left side of the histogram) genes. Genes with log_2_ fold change >10 are indicated. See also Figures S10–S12. The RNA-Seq data are available at Database: NCBI GEO, accession number GSE107622.

RNA sequencing (RNA-seq) analysis of the 12 cell lines identified 508 genes that were significantly differentially expressed (DEG) between PD-L1_IND_ and PD-L1_CON_ cell lines (p value <0.05, FDR corrected and log_2_ fold change of mean FPKM ≥2). Of these DEGs, 222 genes were down-regulated (Figure 3A), whereas the remaining 286 genes were up-regulated in PD-L1_CON_ cells (Figure 3B). Down-regulated genes in PD-L1_CON_ lines were negatively correlated with *CD274* mRNA levels, for which expression was generally higher in PD-L1_CON_ than PD-L1_IND_ cell lines (see the bottom panel of Figure 3A), whereas up-regulated genes were positively correlated with *CD274* mRNA levels. This result suggests that the DEG profile in these cell lines could have a functional role in determining PD-L1 expression status. Up-regulated genes in PD-L1_CON_ cell lines were strongly negatively correlated with global methylation levels, whereas down-regulated genes were positively correlated. We identified a group of 58 genes that showed very high up-regulation in PD-L1_CON_ cell lines (log_2_ fold change >10, Figure 3E, right side of the distribution) compared with 19 genes that showed very strong down-regulation in the PD-L1_CON_ cell lines (Figure 3E, left distribution). The hypomethylated state of the PD-L1_CON_ lines was possibly associated with the up-regulation of global mRNA expression in DEGs, which is conceptually similar to the up-regulation of gene expression upon reduction of DNA methylation levels following DNA methyl transferase (DNMT) inhibitor treatment (DNMTi), as reported in breast, ovarian, and colorectal cancer cell lines (Li et al., 2014).

Functional gene enrichment analysis revealed that the down-regulated genes in PD-L1_CON_ lines were mainly involved in development and cell differentiation. These gene sets were also enriched for the melanin biosynthesis pathway (Figures 3C and S10). In contrast, the up-regulated genes were significantly implicated in several cancer hallmark-related activities, including epithelial-mesenchymal transition (EMT), interferon gamma response, up-regulation of the KRAS signaling pathway, hypoxia, and TNFA signaling mediated by NF-κB. In addition, these genes were highly enriched for cytokine production and signaling pathways (Figures 3D and S11). The most highly up-regulated genes (n = 58) in PD-L1_CON_ cell lines were enriched for T cell differentiation (Figure S12).

### Differential Methylation Affects Specific Genes; *DNMT3A* and *UHRF2* mRNA Expression Is Strongly Associated with Both Global Methylation Levels and *CD274* (PD-L1) mRNA Expression Level Changes

**Figure 4:**
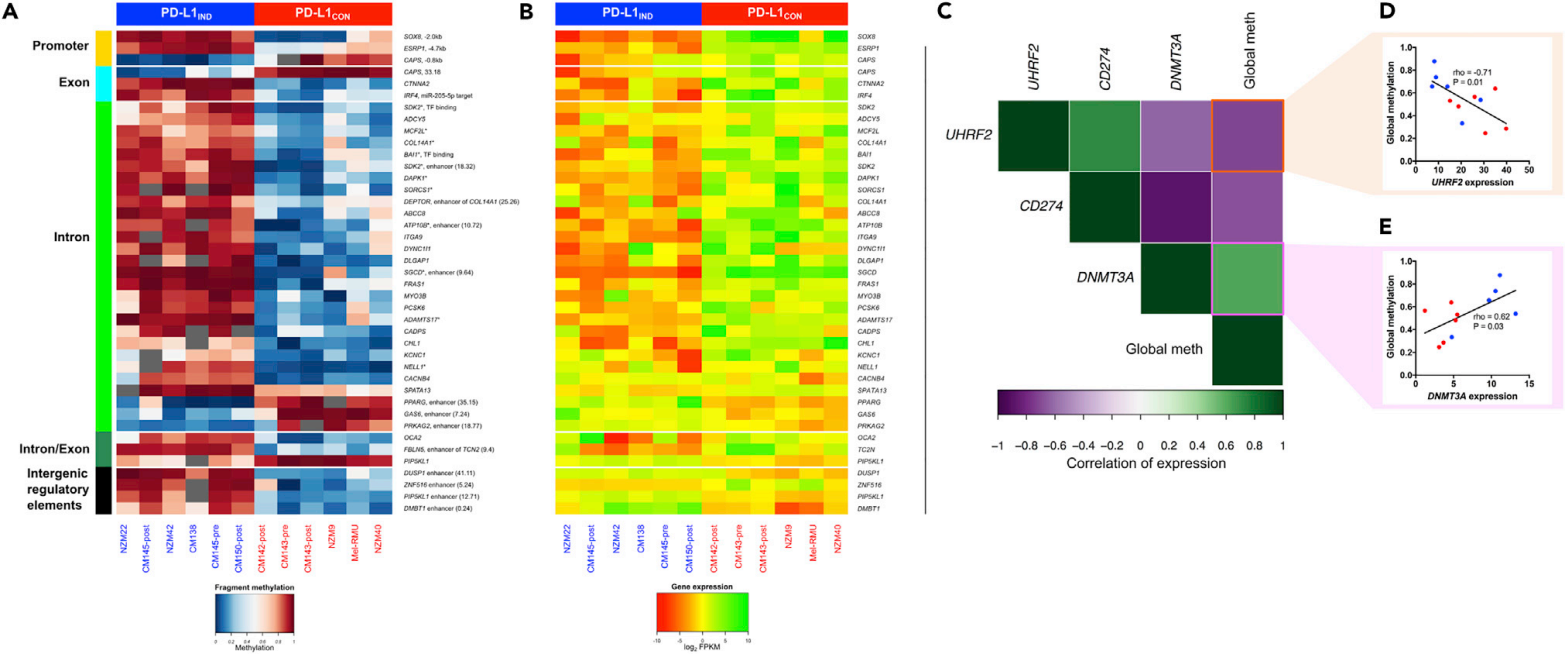
Differential Methylation Pattern and Relationship with Differential Expression in PD-L1_IND_ and PD-L1_CON_ Cell Lines and Role of Epigenetic Regulators (A) Methylation heatmap of PD-L1_CON_ and PD-L1_IND_ cell lines for the differentially methylated fragments (DMFs) in different genomic elements showing the relationship with differential mRNA expression (blue = unmethylated, red = fully methylated). For several genes, multiple DMFs showed a strong correlation and are indicated as *. (B) Mean-centered heatmap of the expression level (log_2_ FPKMs) of the 39 genes that are regulated by methylation levels. (C) Correlogram showing cross-correlation of the key epigenetic regulator genes with *CD274* (PD-L1) expression and global RRBS methylome levels. (D and E) Relationship between mRNA level and RRBS methylome for the analyzed cell lines for *de novo* methylation machinery genes *UHRF2* (D) and *DNMT3A* (E). Spearman rho and statistical significance are shown. See also Figures S13–S15 and Table S5.

Next, we sought to identify specific genes that were potentially directly regulated by DNA methylation. Of the 508 DEGs, 39 genes also harbored differential methylation (Figures 4A and 4B, Table S5). The majority of these DEGs contained gene body DMFs (32 of 39), and only three genes showed differential methylation in the promoter regions (*SOX8, ESRP1*, and *CAPS*). For four genes (*DUSP1, ZNF516, PIP5KL1*, and *DMBT1*) we identified intergenic enhancer-associated DMFs, and loss of methylation in these regions accompanied the overexpression of these genes in PD-L1_CON_ cells. Moreover, loss of gene body methylation in PD-L1_CON_ lines was strongly positively correlated with down-regulation of gene expression and vice versa. This finding is consistent with observations that high gene body methylation is associated with high mRNA expression in the majority of genes (Aran et al., 2011, Bird, 1995, Lister et al., 2009). Several differentially expressed genes were associated with multiple DMFs, particularly in the gene body, consistent with the notion that DNA methylation was involved in regulating their mRNA expression levels in PD-L1_IND_ and PD-L1_CON_ cells (indicated by * in Figure 4A and Table S5).

We took advantage of the methylome and transcriptome data to more closely examine whether widespread global hypomethylation of PD-L1_CON_ cell lines could be explained by the expression of methylation machinery genes (Figure 4C and additional data in Table S6). The *de novo* methylation machinery genes, *DNMT3A* and *UHRF2,* were significantly correlated with global methylome status, as well as *CD274* expression levels. The two methylation machinery genes may promote opposing effects on the RRBS methylome, as *DNMT3A* showed a positive correlation (rho = 0.62, Figure 4E), whereas *UHRF2* (an E3 ubiquitin ligase) was strongly negatively correlated (rho = −0.71, Figure 4D) with global methylation levels. Moreover, *DNMT3A* showed significant negative correlation with *CD274* mRNA expression (rho = 0.88), whereas *UHRF2* was significantly positively correlated with *CD274* expression (rho = −0.73). In addition, these genes exhibited a significant negative correlation with each other in their mRNA profiles (rho = −0.62, p value = 0.03, Figure 4C). We then assessed protein levels using western blots and found that DNMT3A protein levels were correlated with mRNA levels across all cell lines, with a generally higher level of DNMT3A protein in the PD-L1_IND_ lines compared with PD-L1_CON_ lines. However, no consistent differences were observed in UHRF1 and UHRF2 protein levels between the two groups of cell lines (Figures S13 and S14). In addition, neither the methylation maintenance gene, *DNMT1*, nor genes encoding the active demethylating enzymes (*TET1, TET2* and *TET3*) or the deamination enzymes (*APOBEC3F/G*) showed any relationship with the global methylation status or PD-L1 expression (Figure S15).

### PD-L1_CON_ Cell Lines Exhibit Viral Mimicry and an IFN Expression Signature, Similar to that Induced by DNMT Inhibitor Drugs, Which Is a Pattern Also Observed in Melanomas in TCGA

**Figure 5:**
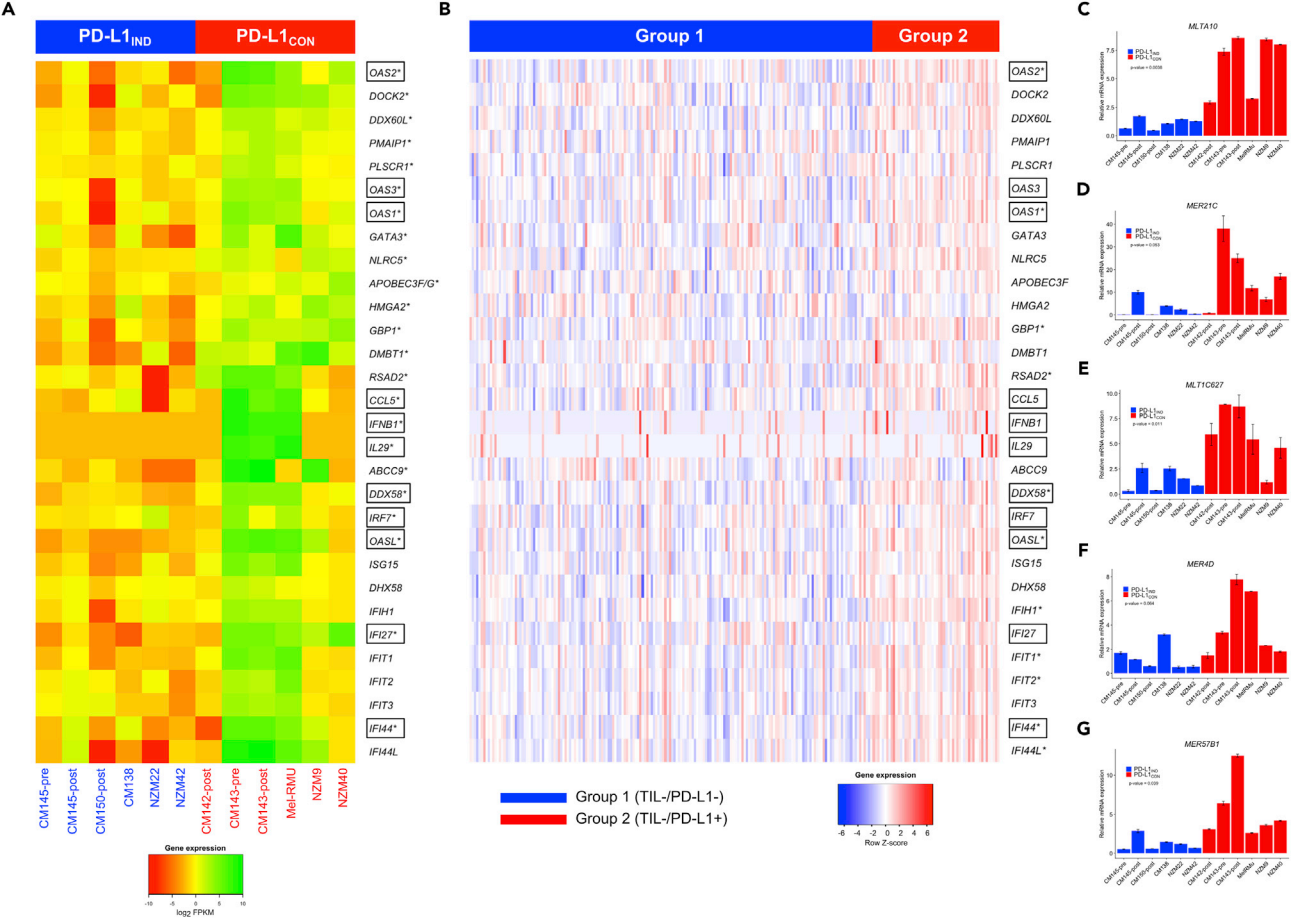
Expression Pattern of Viral Mimicry Genes in PD-L1_IND_ and PD-L1_CON_ Cell Lines and Patients with Melanoma (A) Mean-centered heatmap of the expression level (log_2_ FPKMs) of 30 viral mimicry and immune-system-related genes in PD-L1_IND_ and PD-L1_CON_ cell lines. (B) Heatmap of the expression level (scaled *Z* score) of the same set of 30 genes in TCGA skin cutaneous melanoma data stratified by TIL−/PD-L1− (group 1, representative of inducible in patient group) or TIL−/PD-L1+ (group 2, representative of constitutive in patient group). In both (A) and (B), significantly differentially expressed genes are indicated with an asterisk (*) and the gene names in a box are DNMTi-responsive genes (i.e., previously shown to be silenced and re-expressed upon DNMTi treatment in cancer). (C–G) Gene expression of five of the nine selected ERV genes as measured by RT-qPCR. There is a higher expression of five ERV genes (MLTA10, MER21C, MLT1C627, MER4D, MER57B1) in the PD-L1_CON_ cell lines compared with the PD-L1_IND_ group. Error bars represent SE of two technical replicates. See also Tables S6 and S7.

Demethylation of the cancer genome with DNMTi drugs activates interferon/viral defense, antigen processing, presentation, and host immune genes (Chiappinelli et al., 2015, Li et al., 2014, Liu et al., 2016). In addition, de-repression of these genes is not considered a response to general cellular stress but rather is a specific response to hypomethylation events in the genome. Accordingly, the striking hypomethylation of the PD-L1_CON_ cell lines prompted us to examine whether these patterns were observed in PD-L1_CON_ cells. Significant up-regulation of several type I interferon-stimulated, viral mimicry genes (*IFI44, IFI27, OASL, IL29*) and genes that are upstream of the type I interferon pathway (*IFNB1* and *IRF7*) was observed in PD-L1_CON_ cell lines compared with the PD-L1_IND_ lines (Figure 5A). High innate expression of several genes in PD-L1_CON_ cell lines has been reported to be responsive to DNA-demethylating drugs in other studies (indicated in the box in Figure 5A) (Chiappinelli et al., 2015, Liu et al., 2016). Furthermore, DNMTi treatment has been reported to trigger cytosolic sensing of double-stranded RNA (dsRNA), causing a type I interferon response and apoptosis (Chiappinelli et al., 2015). Consistent with these findings, we observed relatively high mRNA expression of the dsRNA sensor RIG-I (*DDX58*) (the mean log_2_ fold increase in expression of *DDX58* was 4.96 between PD-L1_CON_ and PD-L1_IND_ cell lines compared with the mean log_2_ fold increase in expression of *CD274*, which was 6.11), as well as upstream transcriptional activators, including *IRF1*, in PD-L1_CON_ cell lines. DDX58 induces IFNB1 by signaling through mitochondrial proteins. However, we did not observe a difference in the expression of *TLR3* (another dsRNA sensor). These findings may be related to the innate hypomethylation phenotype in PD-L1_CON_ cells.

To examine if the gene expression patterns we observed in PD-L1_CON_ cell lines were also observed in melanoma tumors, we analyzed RNA-seq data from TCGA-SKCM patients. We used the same group 1 (representative of inducible) and group 2 (representative of constitutive) melanoma cohorts as described in the previous section. Similar to our findings in PD-L1_IND_ and PD-L1_CON_ cell lines, we identified the trend of significant up-regulation of viral mimicry and immune response genes in group 2 patients compared with group 1 patients with melanoma, including 11 viral mimicry genes that were significantly up-regulated in group 2 patients compared with group 1 (after FDR adjustment at 5% and log_2_ fold change of 0.5, indicated as * in Figure 5B).

As these data suggest that global hypomethylation in PD-L1_CON_ melanoma cells induces the viral mimicry pathway, including activation of human endogenous retrovirus (HERV) genes, to explore this further we used RT-qPCR to measure the expression of nine HERV genes that were previously identified to be up-regulated in a colorectal cancer cell line upon DNMTi treatment (Roulois et al., 2015). Indeed, the expression of five of these nine HERV genes was higher in the PD-L1_CON_ cell lines compared with the PD-L1_IND_ cell lines (Figures 5C–5G). This included *MLTA10* (mean-fold increase = 5.8, p value = 0.0038), *MER21C* (mean fold increase = 5.8, p value = 0.053), *MLT1C627* (mean-fold increase = 4.2, p value = 0.011), *MER4D* (mean-fold increase = 3.1, p value = 0.064), and *MTL2B4* (mean-fold increase = 4.4, p value = 0.039). Therefore, these data are consistent with the notion that an innate hypomethylated state is associated with the up-regulation of HERV genes together with the activation of a viral mimicry response and increased PD-L1 levels.

### Global Demethylation with DNMTi Treatment Induces PD-L1 Expression, an Effect that Is Particularly Pronounced in Inducible Melanoma Cell Lines

**Figure 6:**
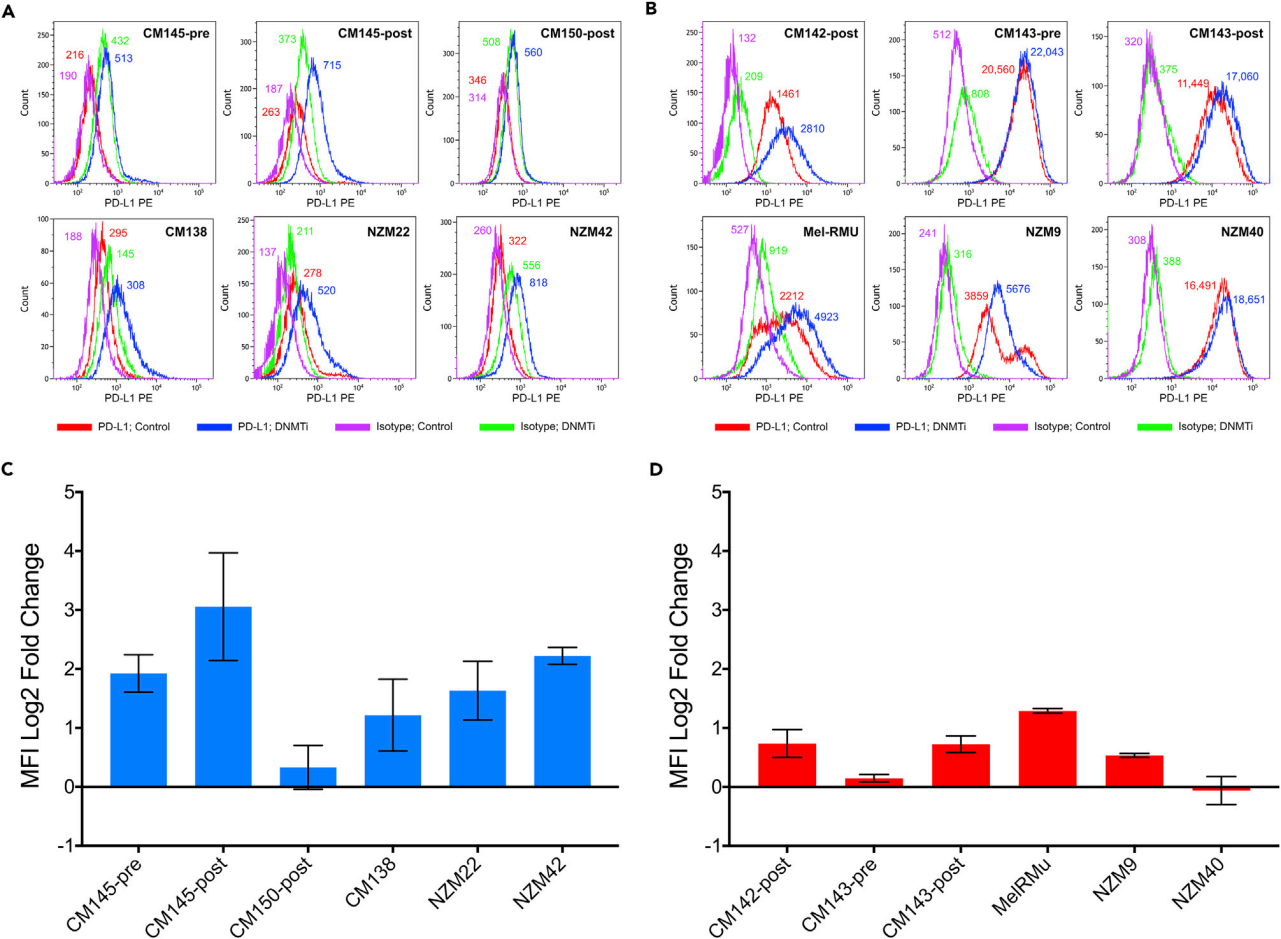
Up-regulation of PD-L1 Cell Surface Expression Upon DNMTi (Demethylation) Treatment in PD-L1_IND_ and PD-L1_CON_ Cell Lines Flow cytometry analysis for PD-L1_IND_ (A) and PD-L1_CON_ (B) cell lines was performed at day 6 following 3 days treatment with decitabine (DNMTi; 0.5μM) or mock treatment (DMSO). Changes of PD-L1 expression between DNMTi treated and the control for PD-L1_IND_ (C) and PD-L1_CON_ (D) cell lines were calculated using medium fluorescence intensities (MFI) and the formula log_2_ ([(MFI_antibody, treated_)−(MFI_isotype, treated_)]/[(MFI_antibody, mock_)−(MFI_isotype, mock_)]) (Wrangle et al., 2013). Error bars represent SE of two technical replicates. See also Figures S16 and S17.

Finally, we hypothesized that, if global hypomethylation regulates PD-L1 expression, then reducing genomic methylation levels will lead to enhanced PD-L1 expression, particularly in the inducible lines (PD-L1_IND_), as they exhibited higher genomic methylation levels. We treated 12 melanoma cell lines with decitabine (DNMTi treatment causing global demethylation) and observed up-regulation of cell surface PD-L1 expression upon DNMTi treatment in all 12 cell lines, although the degree of up-regulation varied. Inducible lines showed stronger induction and higher up-regulation of PD-L1 expression upon demethylation compared with constitutive lines (Figures 6A–6D). In five of the six inducible cell lines, cell surface PD-L1 levels were up-regulated >2 fold and particularly in CM145-pre, CM145-post, and NZM42, which showed an average fold increase of 3.9, 10.0, and 4.7, respectively (Figure 6C). The PD-L1_CON_ lines also generally showed PD-L1 up-regulation. However, as they were already expressing high levels of PD-L1, the degree of change was relatively small compared with that of the inducible lines (Figures 6C and 6D). Among the PD-L1_CON_ lines, only Mel-RMU showed >2-fold (2.44) up-regulation of PD-L1 upon demethylation (Figure 6D). These patterns were true for low (100 nM) and moderate (500 nM) doses of decitabine. By western blot, total PD-L1 protein levels were up-regulated by decitabine treatment in two PD-L1_CON_ lines, although in PD-L1_IND_ lines, despite observing an increase in *CD274* mRNA levels after decitabine treatment (data not shown) and an increase in cell surface PD-L1 levels (see Figure 1), total PD-L1 protein levels were not generally increased upon decitabine treatment, which may be due to the relatively high levels of decitabine-induced cell death in these cells (Figure S16). In addition, we treated these cell lines with vitamin C, which promotes viral mimicry (Liu et al., 2016) and induces active demethylation by enhancing TET enzyme activity; however, this treatment did not result in any significant further increase of cell surface PD-L1 expression in either the inducible or constitutive lines, except in the inducible cell line CM145-post, which showed a 2.06 average fold increase in PD-L1 upon vitamin C treatment (Figure S17).

## Discussion

The success of immunotherapy, based on the inhibition of the PD1 checkpoint in lymphocytes with mAbs against PD1 or PD-L1, has focused attention on the regulation of PD-L1 expression on cancer cells. In previous investigations, we and others have defined many of the mechanisms involved in inducible PD-L1 expression on melanoma but we have not previously defined the basis for constitutive forms of PD-L1 expression (Gowrishankar et al., 2015, Madore et al., 2016). In the present study, we have examined whether DNA methylation plays a functional role in the regulation of constitutive PD-L1 expression by using sequencing-based genome-wide DNA methylation profiling combined with whole transcriptome profiling.

### Constitutive PD-L1 Expression in Melanoma Is Associated with Global Hypomethylation and Transcriptomic Up-Regulation

Using RRBS genome-wide methylation profiling we identified a striking global loss of methylation between cell lines with constitutive PD-L1 expression, compared to inducible PD-L1 lines. The large methylation differences, which were distinctly identified by RRBS analysis, were confined mainly to intergenic and intronic regions, rather than promoter regions. In contrast, the same pattern of methylation differences could not be detected in the TCGA 450K melanoma methylation data, which we argue is because probes in the 450K platform were mainly located in gene promoters, whereas relatively few 450K probes were located in the gene body or intergenic regions. By RRBS analysis many genomic regions exhibiting strongly divergent methylation patterns were identified between the inducible and constitutive cell lines. These RRBS methylation patterns were remarkably similar within a group (inducible or constitutive), which we hypothesize is the result of a unified mechanism leading to hypomethylation of particular genomic regions across all the PD-L1 constitutively expressing cell lines. Consistent with previously reported data, we found the *CD274* promoter was unmethylated (Chatterjee et al., 2016), with no evidence of differential methylation occurring in the core promoter itself, which argues against methylation of the *CD274* promoter being involved in constitutive PD-L1 expression. In addition, blocking IFN-γ or interferon type I or type II with antibodies did not inhibit constitutive PD-L1 expression but (surprisingly) consistently enhanced constitutive PD-L1 expression. As expected, the same treatment with IFN-γ-blocking antibodies was able to strongly suppress interferon-driven PD-L1 induction in PD-L1_IND_ cell lines. These results argue against the notion of autocrine interferon-dependent regulation of PD-L1 expression in PD-L1_CON_ cells.

Transcriptome profiles in these cell lines correlated with global methylome status, and DEGs were highly up-regulated in the constitutive lines, consistent with their genomic hypomethylated state. Up-regulated genes in the PD-L1_CON_ lines were associated with EMT, KRAS signaling, hypoxia, and NF-κB signaling, consistent with the known key pathways that regulate PD-L1 transcription (Chen et al., 2016). The constitutive up-regulation of these pathways as a result of global hypomethylation could be a causative factor associated with constitutive PD-L1 expression.

Of 557 differentially expressed genes, only 39 showed significant changes in methylation, with methylation differences mainly occurring in the gene body or enhancer regions associated with these genes. This suggests that, rather than promoter methylation differences, large genomic methylation differences outside the promoter regions could play a regulatory role in promoting the constitutive versus inducible PD-L1 expression. Interestingly, although global hypomethylation was correlated with mRNA up-regulation in PD-L1_CON_ lines, for the majority of candidate genes, loss of gene body methylation was linked with their down-regulation in the PD-L1_CON_ cell lines. This analysis identified methylation in several candidates that have the potential to regulate PD-L1 and therefore potentially could play a role in melanoma biology. For example, *IRF4, ESPR1*, and *DAPK1* showed notable down-regulation of expression and loss of gene body methylation in PD-L1_CON_ cell lines. *IRF4* is upstream of the PD-L1 signaling pathway, and reduced levels of the IRF4 transcription factor lead to up-regulation of PD-L1 expression and promote T cell dysfunction (Wu et al., 2017). In addition, in TCGA patients with melanoma, reduced levels of *ESRP1* (which encodes a master splicing regulator involved in EMT) were correlated with increased immune checkpoint expression (PD-L1 and CTLA4) and elevated tumor-associated immune cytolytic activity (Yao et al., 2016). In addition, reduced levels of *DAPK1* were shown to be associated with reduced sensitivity to BRAF inhibitor therapy, suggesting its possible role in targeted melanoma therapy (Xie et al., 2017). These candidates and other genes containing multiple DMFs and deregulated gene expression warrant future investigation in the context of PD-L1 to elucidate their mechanistic specific role.

### Reduced Expression of DNMT3A Correlates with Global Hypomethylation in PD-L1 Constitutive Melanomas

Global hypomethylation is perhaps the most accepted epigenetic paradigm in cancer (Baylin and Jones, 2011), yet the mechanisms involved in this are not completely clear. Our study offers fresh insights into the possible mechanisms for global hypomethylation in melanoma cell lines. The *de novo* methylating enzyme DNMT3A was strongly negatively correlated at mRNA and protein levels with PD-L1 expression and was positively associated with globally elevated methylation levels, which is consistent with the notion that hypomethylation in PD-L1_CON_ cell lines may be the result of reduced levels of DNMT3A. Additional investigations are required to determine whether DNMT3A indeed plays a central role in the global hypomethylation and levels of PD-L1 expression in PD-L1_CON_ cells.

We additionally observed that mRNA expression of *UHRF2*, an E3 ligase that degrades DNMT3A, was positively correlated with *CD274* levels and negatively correlated with *DNMT3A* expression and global methylation. UHRF2 has been previously reported to be a potential mediator of global hypomethylation (Jia et al., 2016), and a recent study indicated that UHRF1 and UHRF2 negatively regulate *de novo* DNA methylation by promoting DNMT3A degradation (Jia et al., 2016). UHRF2 protein expression levels in our western blots were not correlated with changes in global methylation or PD-L1 expression, although additional investigations of the relationship between UHRF2, DNMT3A, PD-L1, and global hypomethylation in melanoma may still be warranted.

Two other mechanisms that could potentially drive global hypomethylation in melanoma are direct methylation deamination (by the deaminase family gene *APOBEC* [Cortellino et al., 2011, Kumar et al., 2014]) and active removal of methylation via TET enzymes (Guo et al., 2011, Ito et al., 2011). Expression of the *APOBEC3F/G* gene was positively correlated with *CD274* expression; however, it was not strongly related with global methylation levels. Furthermore, active demethylation by TET enzymes did not appear to be involved in the PD-L1_CON_ DNA methylation levels, as expression patterns of the TET family genes were not correlated with global methylation levels or *CD274* expression. This is also consistent with our experimental data whereby treatment with vitamin C failed to induce significant up-regulation of cell surface PD-L1 expression, whereas DNMTi treatment significantly increased cell surface PD-L1 levels. Taken together, our data provide evidence that *de novo* regulation of global methylation levels in melanoma is potentially part of the mechanisms regulating PD-L1 expression.

We identified, in addition to methylation of regulatory genes, several chromatin-modifying factors (e.g., *KDM4C* and *PRDM* family genes), histone-modifying genes (e.g., *CDK9, HMG20B*), and chromatin-remodeling genes (*INO80*) that were associated with PD-L1 expression and global methylome status. A recent study demonstrated transcriptional rewiring resulting from chromatin remodeling in exhausted T cells following PD-L1 blockade (Pauken et al., 2016). This epigenetic rewiring resulted in their failure to become memory T cells in the presence of high antigen levels. Moreover, evidence for global chromatin remodeling during melanoma progression is emerging (Fiziev et al., 2017). For example, *INO80* was recently shown to reduce nucleosome occupancy and promote oncogenic transcription in melanoma (Zhou et al., 2016). It is plausible that these genes contribute to differential chromatin remodeling and lead to alterations in the epigenetic landscape in PD-L1_IND_ and PD-L1_CON_ cell lines, and our data provide a basis for exploring the role of chromatin and histone changes in determining PD-L1 expression in tumors.

We reasoned that one possible explanation for the association between the global hypomethylation and constitutive PD-L1 expression is that global hypomethylation results in changes in the signaling pathways involved in immune response and generation of a constant “on” signal. For example, this could include the hypomethylation and activation of stimulator of IFN genes (STING), an adaptor protein associated with the ER (Corrales et al., 2016, Corrales et al., 2017). STING was reported to be defective in many types of cancer. In colon cancer, for example, this results from methylation of genes encoding STING or other proteins in the pathway (Xia et al., 2016a). Similar studies in melanoma cell lines have shown loss of STING in 3/11 lines and of cGAS (the synthase generating cyclic dinucleotides) in 4/11 lines. Immunohistochemical (IHC) studies showed that over 40% of metastases lacked STING or cGAS expression (Xia et al., 2016b). Proteins downstream of STING, such as IRF3 and NF-κB, were also lost in some cell lines. STING is not involved in the response to dsRNA. Nevertheless, cGAS has been reported to be essential for the response to immune checkpoint blockade in B16 mouse melanoma studies (Wang et al., 2017).

### DNMTi Treatment Increases PD-L1 Expression, Which like Constitutive PD-L1 Expression in Melanoma, Is Associated with a Viral Mimicry Phenotype

Studies in epithelial cancers have shown that treatment with DNMTi can induce an IFN gene signature response in the cells, including PD-L1 (Li et al., 2014). The latter was suggested to be due to viral mimicry (Roulois et al., 2015) resulting from demethylation of human endogenous retroviral sequences (HERVs), which were then recognized by dsRNA sensors *TLR3*, *MGA5*, and *RIGI* in the cells (Chiappinelli et al., 2015, Chiappinelli et al., 2016). This is consistent with our findings that repeat elements, particularly the HERVs, were strongly hypomethylated in the PD-L1_CON_ cells and that their mRNA expression levels were increased, higher in PD-L1_CON_ than in PD-L1_IND_ cells. Moreover, the hypomethylation phenotype was associated with the up-regulation of genes responsible for generating endogenous immune responses in cancer cells treated with DNMTi, such as several type I interferon-stimulated, viral mimicry-related genes (*IFI44, IFI27, OASL, IL29*) and genes that are upstream of the type I interferon pathway (*IFNB1*, *IRF7*) in PD-L1_CON_ cell lines (Chiappinelli et al., 2015, Liu et al., 2016). Analysis of TCGA melanoma patient transcriptome data revealed that these viral mimicry genes were significantly differentially expressed between constitutive (group 2) and inducible (group 1) patient groups. Taken together, these results support the notion that up-regulation of a viral mimicry phenotype is the result of global hypomethylation in PD-L1_CON_ cells. Further studies are needed to confirm whether constitutive up-regulation of the type 1 IFN pathway is a result of a hypomethylation-mediated viral mimicry phenotype.

In summary, based on our results, we conclude that constitutive expression of PD-L1 is a consequence of global hypomethylation and that global DNA methylation status is an important factor in the regulation of PD-L1. The exact mechanism of how the hypomethylated state regulates pathways involved in PD-L1 expression in melanoma needs to be further investigated, but constitutive expression of PD-L1 in melanoma cells may identify melanomas that have endogenous activation of IFN signaling pathways, analogous to effects of treatment with inhibitors of DNMT enzymes. We identified that down-regulation of *DNMT3A* was associated with global hypomethylation and PD-L1 expression. Further studies are needed to examine whether this subset of melanomas have similar responses to PD1 checkpoint inhibitor treatments or if they require combination therapies that target other consequences of hypomethylation, such as activation of epithelial mesenchymal transition, NF-κB, or hypoxia pathways.

## Methods

All methods can be found in the accompanying Transparent Methods supplemental file

## Data and Software Availability

DNA methylation and transcriptomic data for PD-L1_CON_ and PDL1_IND_ cell lines are available at Database: NCBI GEO, accession number GSE107622.

## Supplemental Information

Document S1. Transparent Methods, Figures S1–S17, and Tables S1–S5 and S7

Table S6. Correlation of Endogenous Retrovirus Genes with CD274 (PD-L1) mRNA Expression and Global DNA Methylation Levels, Related to Figure 4
