# Supplementary material for: Marked Global DNA Hypomethylation Is Associated with Constitutive PD-L1 Expression in Melanoma
Source: iScience. 2018 Jun 28;4:312–25. doi: 10.1016/j.isci.2018.05.021 (PMC6147024; doi:10.1016/j.isci.2018.05.021)
Supplement: Document S1. Transparent Methods, Figures S1–S17, and Tables S1–S5 and S7 [file mmc1.pdf]

ISCI, Volume *4*

Supplemental Information

Marked Global DNA Hypomethylation

Is Associated with Constitutive

PD-L1 Expression in Melanoma

Aniruddha Chatterjee, Euan J. Rodger, Antonio Ahn, Peter A. Stockwell, Matthew Parry, Jyoti Motwani, Stuart J. Gallagher, Elena Shklovskaya, Jessamy Tiffen, Michael R. Eccles, and Peter Hersey

Supplemental Figures:

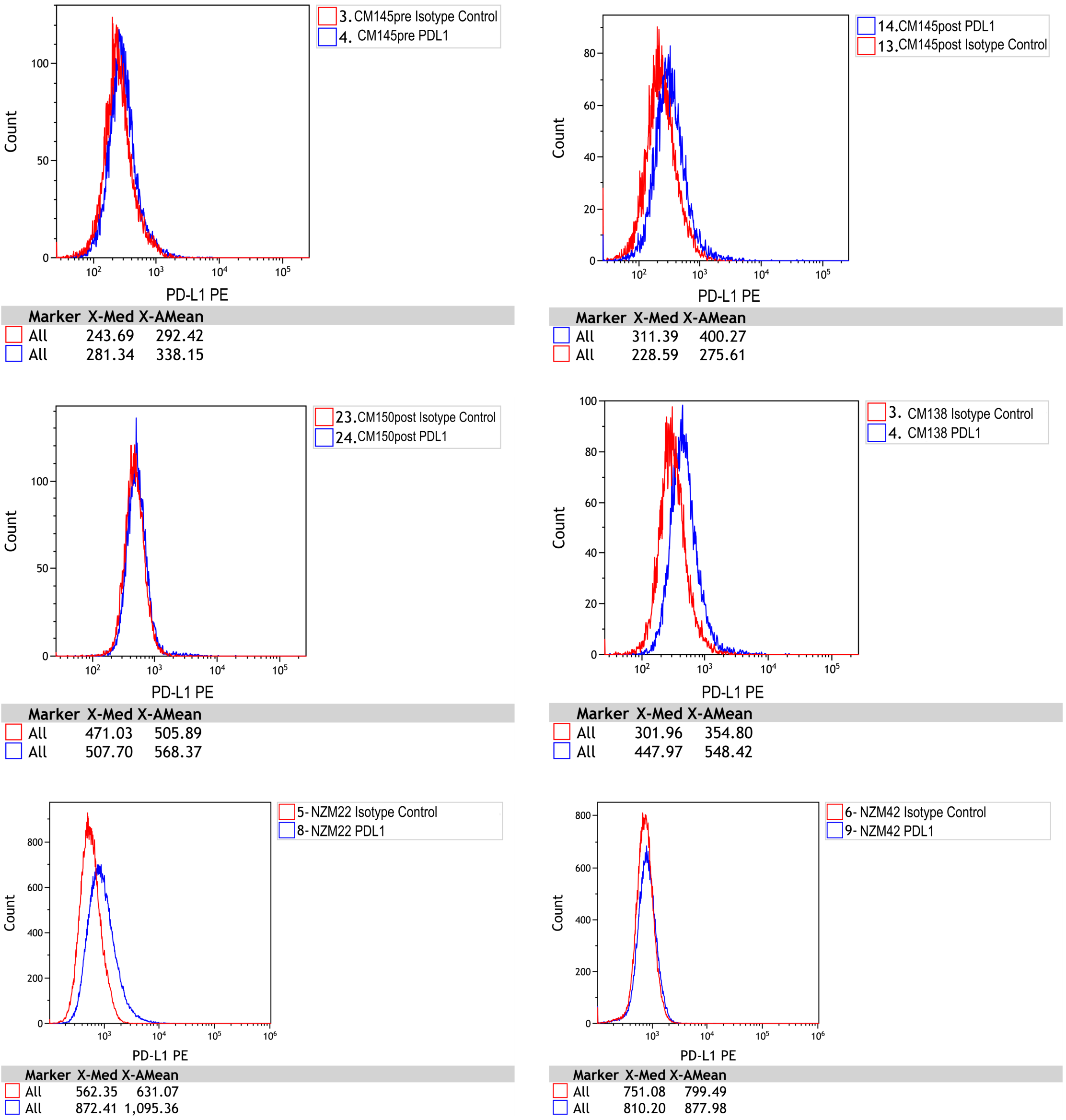

Figure S1. FACS analysis results of the PD-L1$_{IND}$ cell lines. Related to Figure 1. Flow cytometry was used to determine cell surface expression of PD-L1 in the PD-L1$_{IND}$ cell lines. The melanoma cell lines were stained with anti-PD-L1 (PE) and the isotype control antibody.

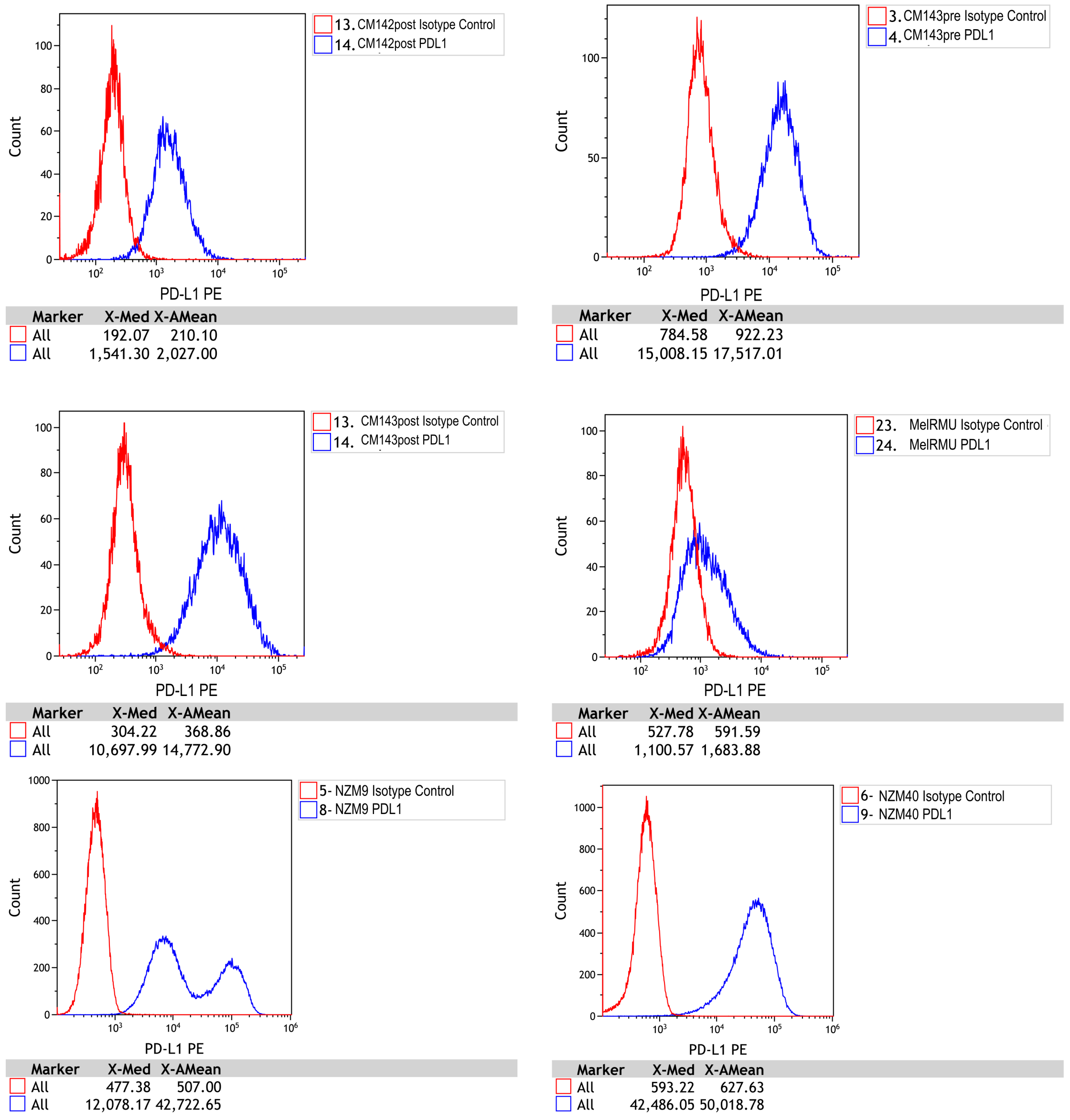

Figure S2. FACS analysis results of the PD-L1$_{CON}$ cell lines. Related to Figure 1. Flow cytometry was used to determine cell surface expression of PD-L1 in the PD-L1$_{CON}$ cell lines. The melanoma cell lines were stained with anti-PD-L1 (PE) and the isotype control antibody. An expression level of at least five-fold higher than the isotype control was considered as constitutive expression of PD-L1.

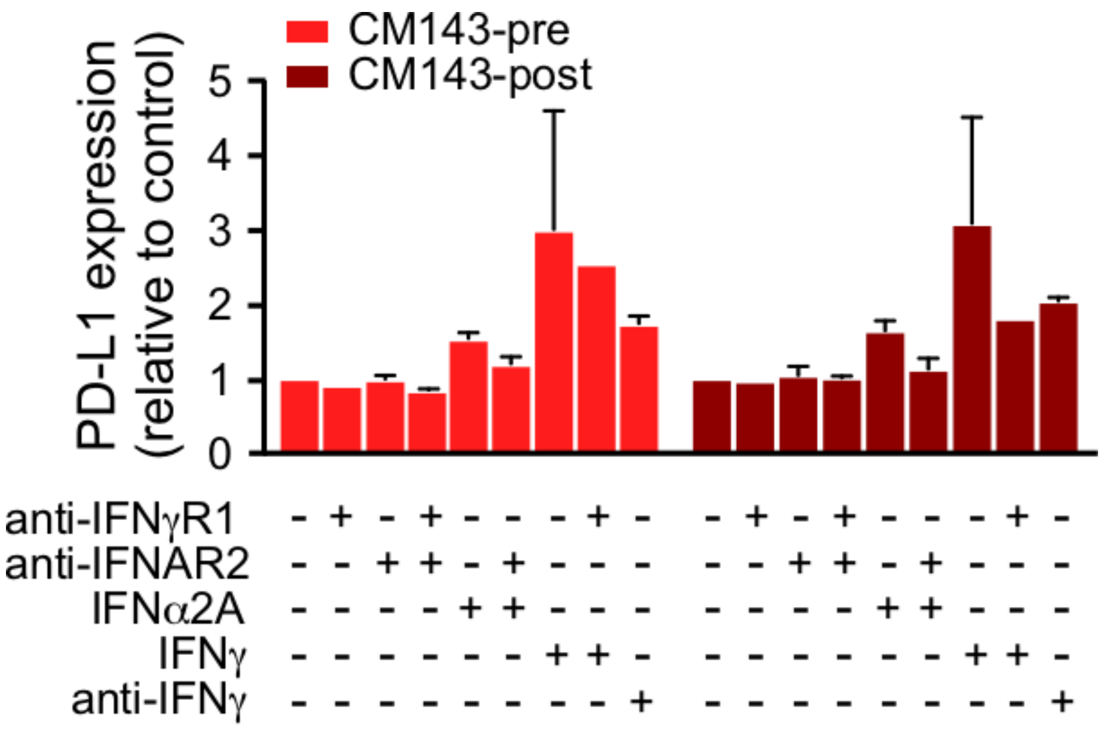

Figure S3. Blocking interferon signalling in PD-L1$_{CON}$ lines does not reduce constitutive PD-L1 expression. Related to Figure 1. Interferon-type I or -type II signaling was blocked with the indicated antibodies in the absence or presence of IFNα2A or 100ng/ml IFNγ as indicated. PD-L1 expression was measured by flow cytometry on day 3. Results are expressed relative to control (no treatment) levels. Error bars represent SE of two technical replicates.

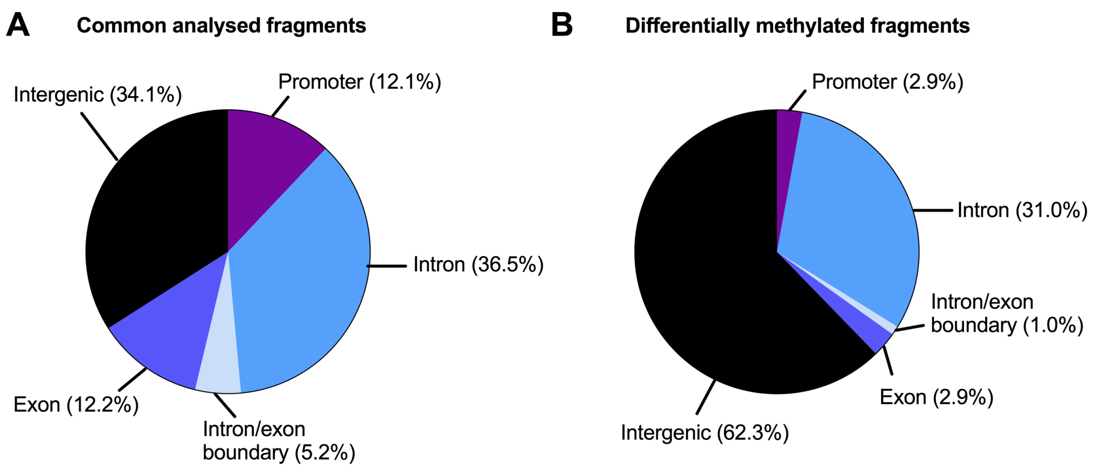

Figure S4. Genomic distribution of the analysed RRBS fragments (A) and the identified DMFs between PD-L1$_{CON}$ and PD-L1$_{IND}$ melanoma cell lines. Related to Figure 2. Gene promoters were defined as -5 to +1 kb from the TSS.

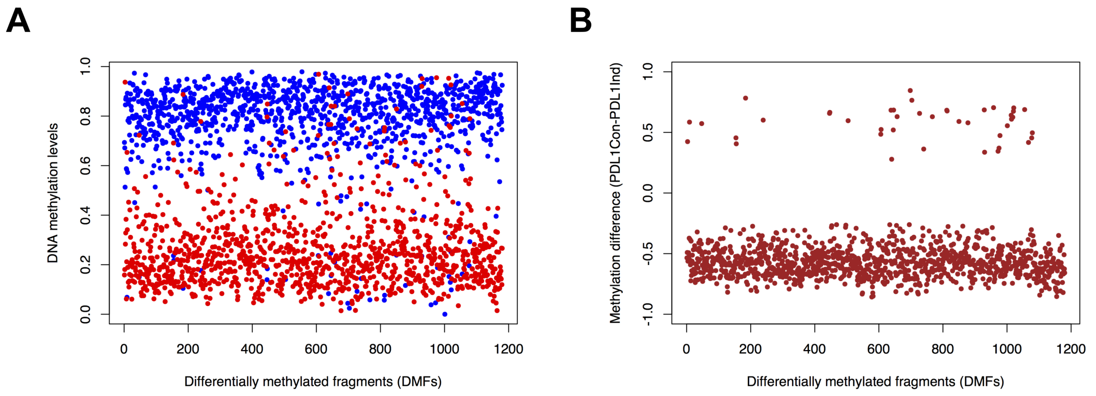

Figure S5. DNA methylation patterns (A) and mean methylation difference (B) for the identified DMFs between PD-L1$_{CON}$ and PD-L1$_{IND}$ melanoma cell lines. Related to Figure 2. All these fragments had high coverage methylation data after filtering for coverage (at least four cell lines in each group had 10 or more reads and at least 2 CpG sites in a fragment. The methylation was shown in a scale of 0 to 1.0 (i.e., 0 to 100%).

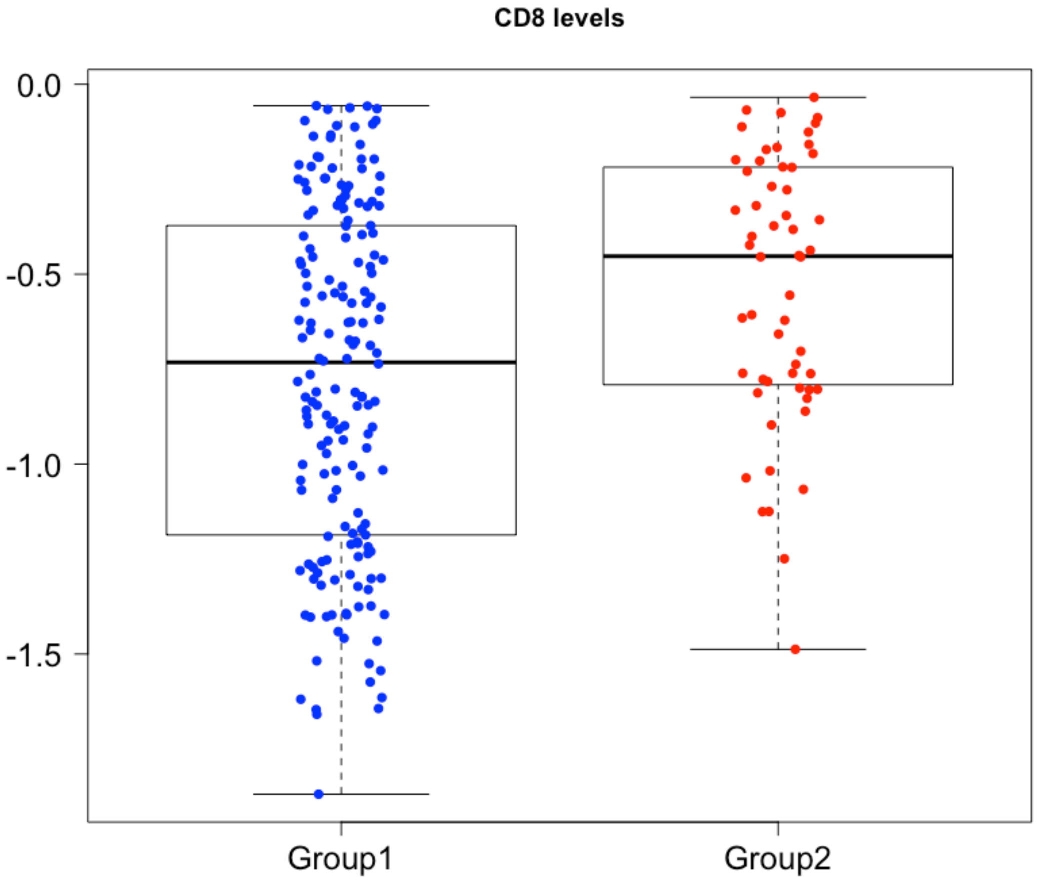

Figure S6. CD8 levels (TILs) based on immune cell deconvolution analysis on TCGA-SKCM RNA-Seq data for Group 1 and Group 2 patients (representative of PD-L1$_{IND}$ and PD-L1$_{CON}$ groups). The boxplots show the median and interquartile range. Related to Figure 2.

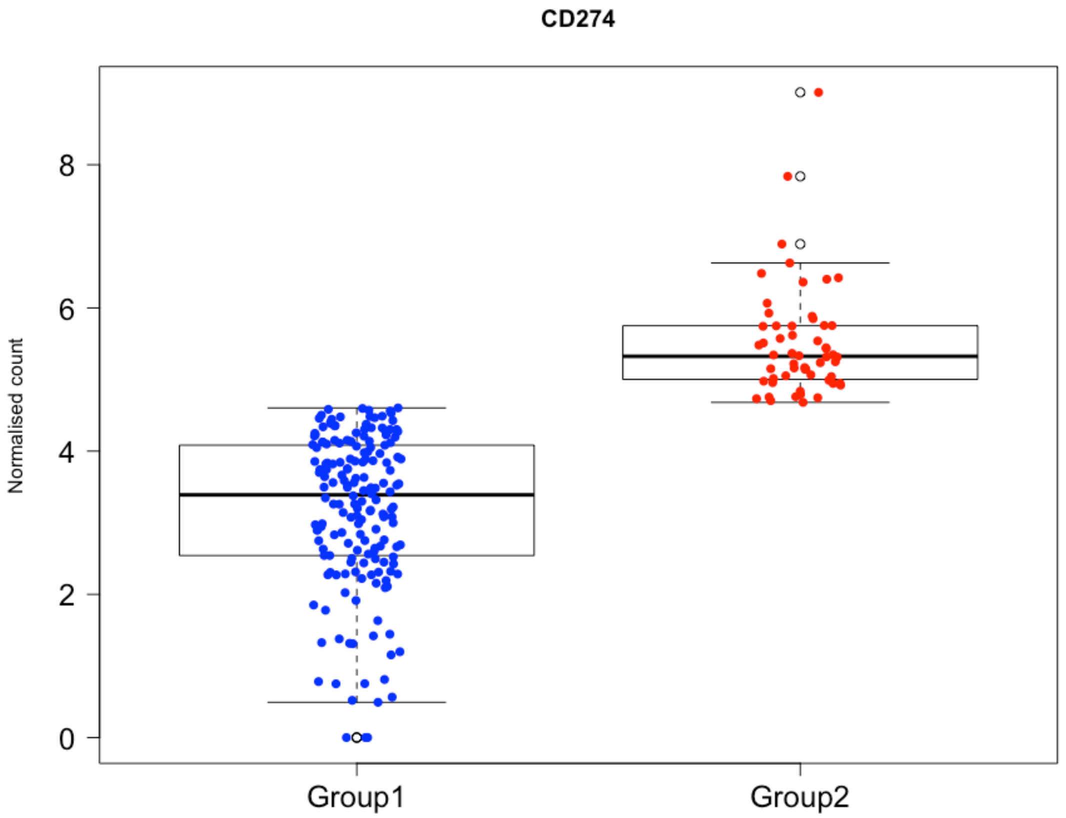

Figure S7. PD-L1 (*CD274*) mRNA levels based on TCGA-SKCM RNA-Seq data for Group 1 and Group 2 patients (representative of PD-L1$_{IND}$ and PD-L1$_{CON}$ groups). The boxplots show the median and interquartile range. Related to Figure 2.

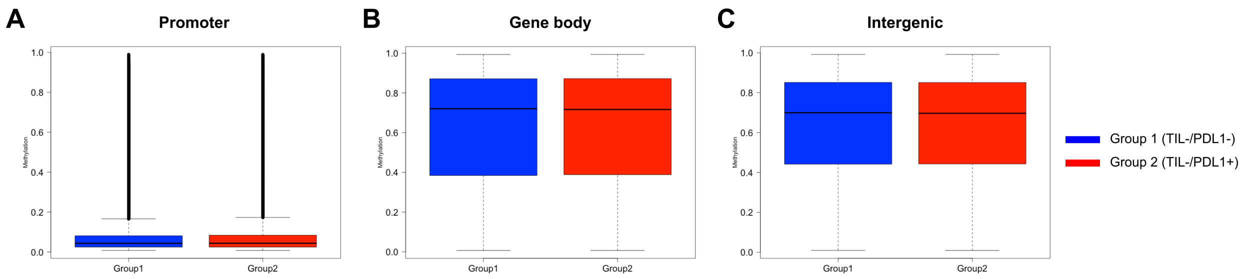

Figure S8. Methylation levels based on 450K TCGA-SKCM data for Group 1 and Group 2 patients (representative of PD-L1$_{IND}$ and PD-L1$_{CON}$ groups). Boxplots show the average beta values and interquartile range of all probes belonging to the feature. Related to Figure 2.

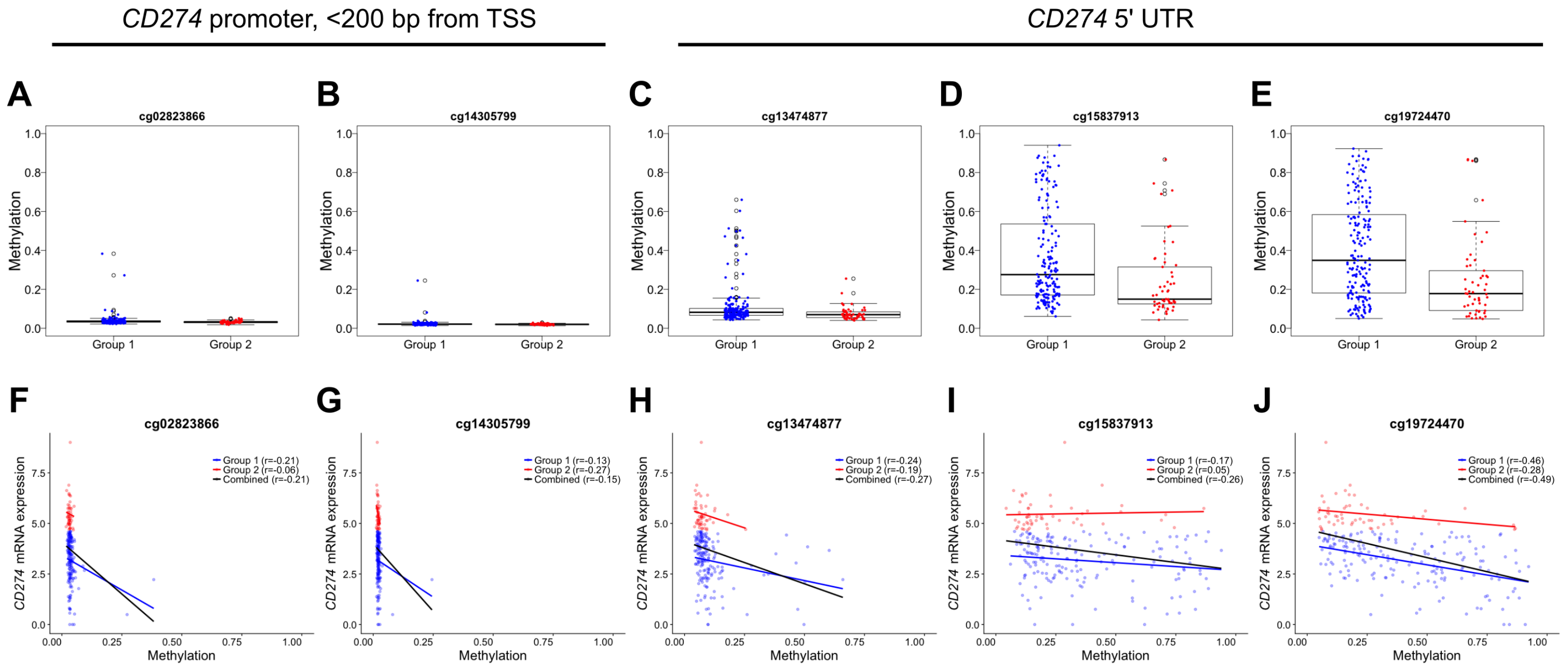

Figure S9. Methylation levels based on 450K TCGA-SKCM data for cg02823866 and cg14305799 probes for Group 1 and Group 2 patients (representative of PD-L1$_{IND}$ and PD-L1$_{CON}$ groups). Related to Figure 2. We specifically analyzed tumours that were TIL-ve to reduce the impact of immune cell signaling on tumour PD-L1 expression, and divided these tumours into Group 1 (PD-L1-ve, n= 180) and Group 2 (PD-L1+ve, n= 54). We considered these two groups (Group 1 and Group 2) as being the most representative of our analysed cell lines (PD-L1$_{IND}$ and PD-L1$_{CON}$, respectively).

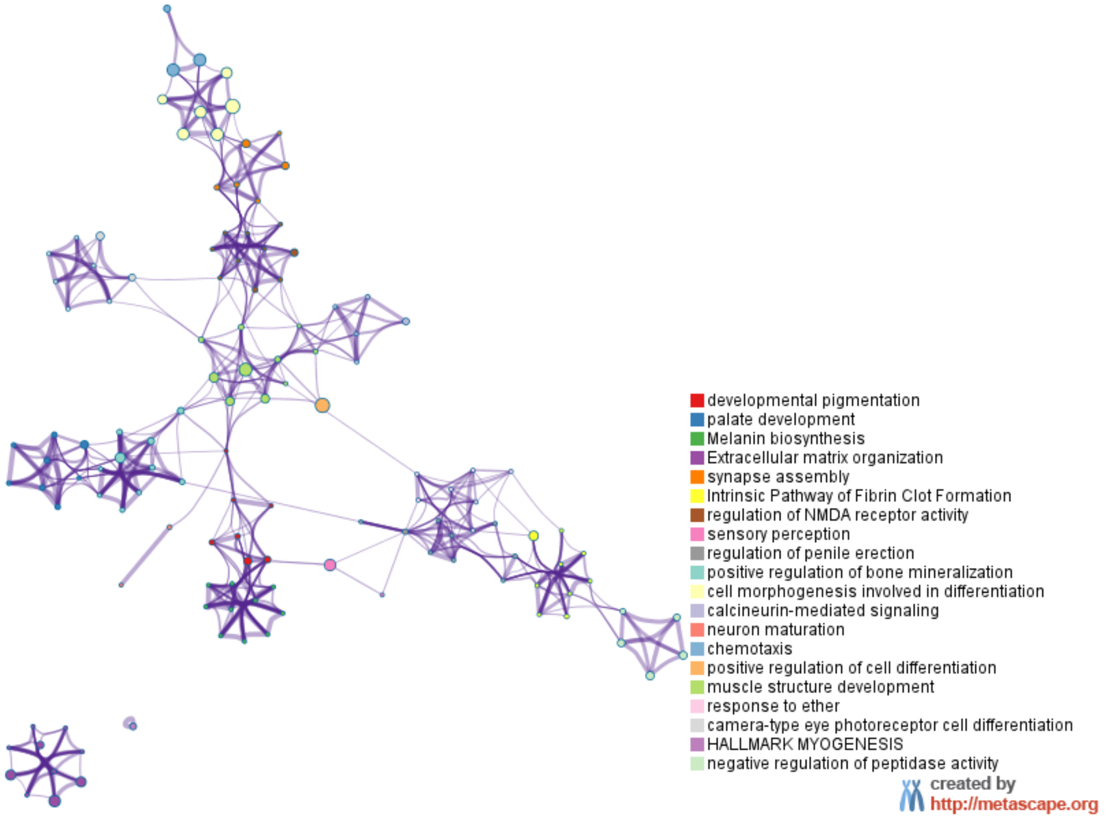

Figure S10. Enrichment network of the 222 genes that are down-regulated in PD-L1$_{CON}$ cell lines. Related to Figure 3. Representative terms from this the GO cluster analysis were converted into a network layout. Each term is represented by a circle node, where the size is proportional to the number of input genes that fall into the particular term, and the color represents its cluster identity (i.e., nodes of the same color belong to the same cluster). Terms with a similarity score > 0.3 are linked by an edge (the thickness of the edge represents the similarity score). The network is visualized with Cytoscape with the default layout. This analysis was performed using Metascape (http://metascape.org/gp/index.html#/main/step1).

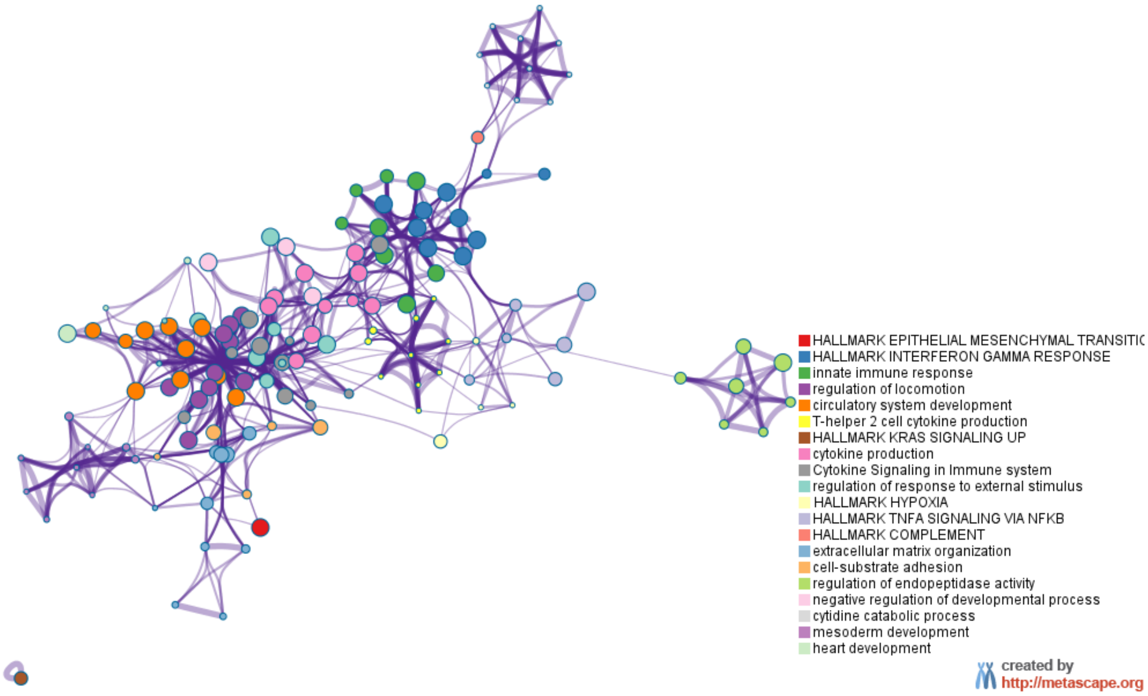

Figure S11. Enrichment network of the 286 genes that are up-regulated in PD-L1$_{CON}$ cell lines. Related to Figure 3. Representative terms from this the GO cluster analysis were converted into a network layout. Each term is represented by a circle node, where the size is proportional to the number of input genes that fall into the particular term, and the color represents its cluster identity (i.e., nodes of the same color belong to the same cluster). Terms with a similarity score > 0.3 are linked by an edge (the thickness of the edge represents the similarity score). The network is visualized with Cytoscape with the default layout. This analysis was performed using Metascape (http://metascape.org/gp/index.html#/main/step1).

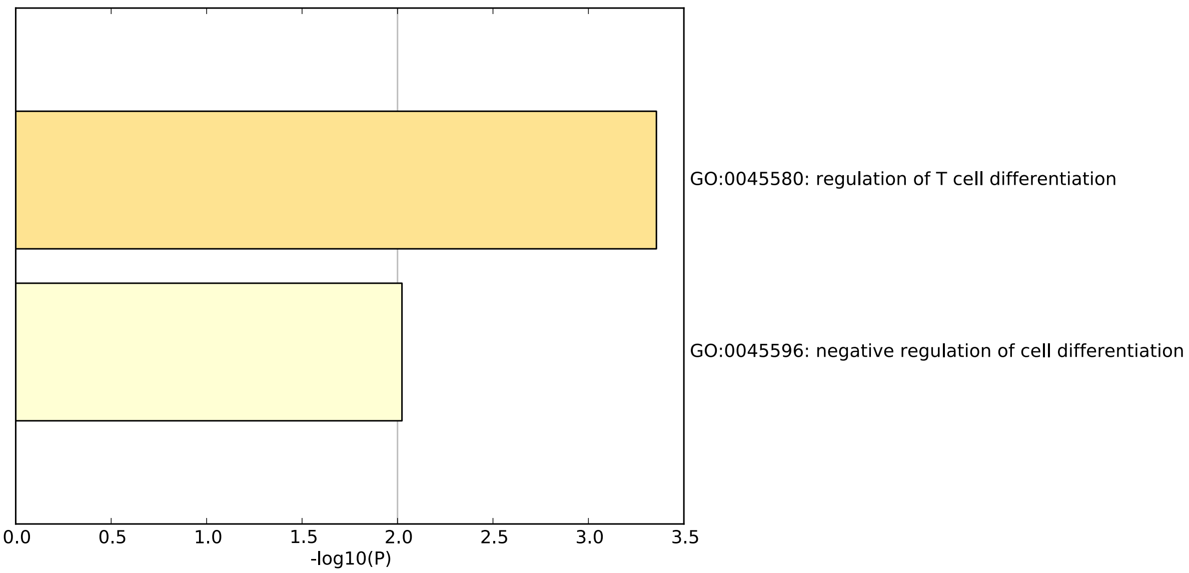

Figure S12. Enriched GO terms relative to the 58 genes that were very highly up-regulated in PD-L1$_{CON}$ cell lines. Related to Figure 3. The x-axis represents –log 10 of the *P*-value. This analysis was performed using Metascape (http://metascape.org/gp/index.html#/main/step1).

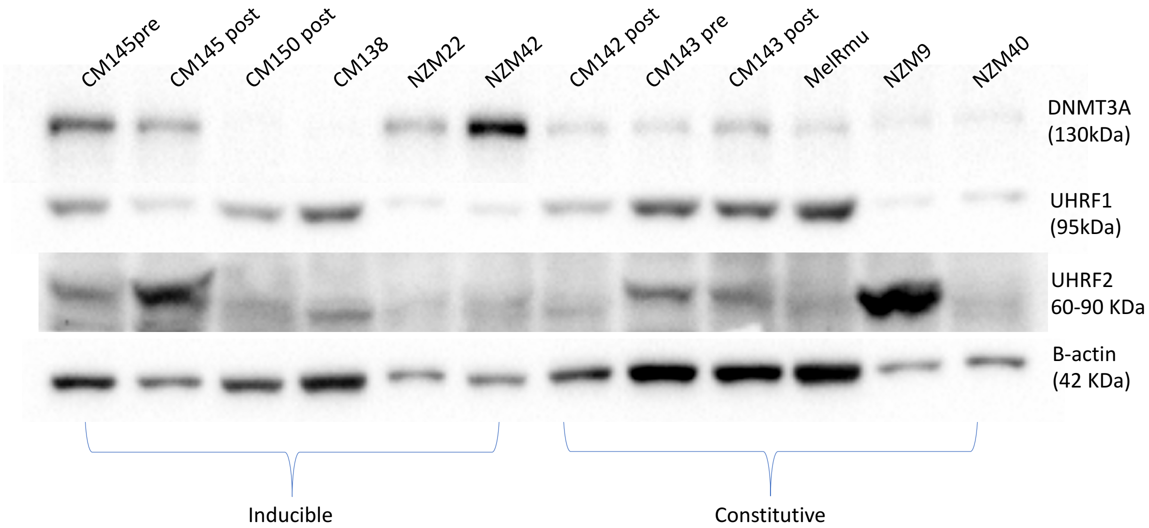

Figure S13. Western blot analysis of DNMT3A, UHRF1 and UHRF2 for PD-L1$_{IND}$ and PD-L1$_{CON}$ groups. Related to Figure 4. Western blots of cell lysates were performed with DNMT3A, UHRF1 and UHRF2 antibodies using a beta-actin antibody as a loading control; representative blots are shown.

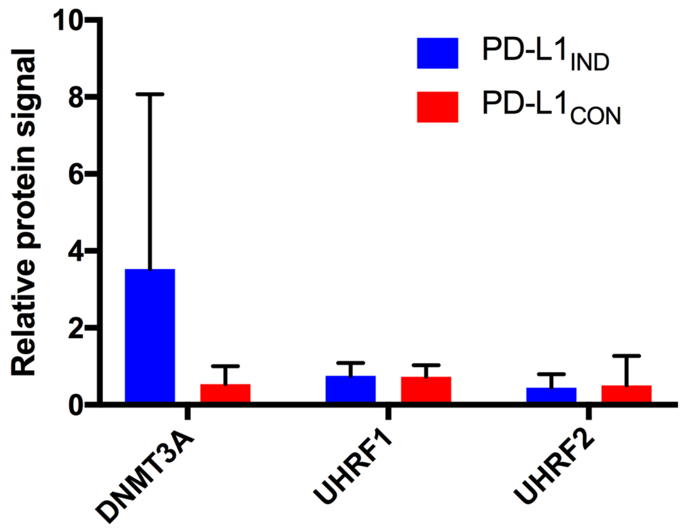

Figure S14. Quantification of DNMT3A, UHRF1 and UHRF2 protein levels for PD-L1$_{IND}$ and PD-L1$_{CON}$ groups. Related to Figure 4. Protein levels of DNMT3A, UHRF1 and UHRF2 were normalized against actin and are shown as mean ± SD for the PD-L1$_{IND}$ (n=6) and PD-L1$_{CON}$ (n=6) groups run in duplicate.

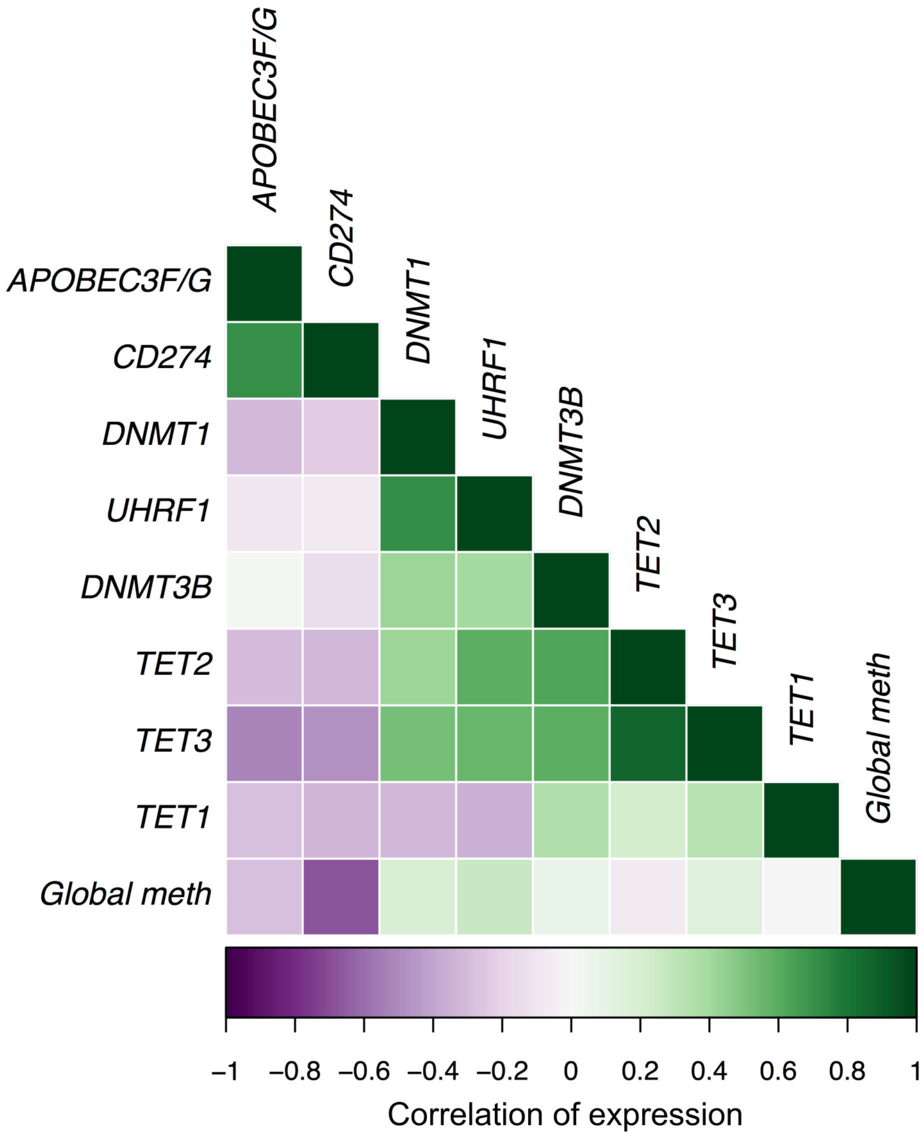

Figure S15. Relationship of methylation regulator genes with PD-L1 expression and global methylation levels. Related to Figure 4. Correlogram showing cross-correlation of major methylation regulator genes with *CD274* expression and global RRBS methylome in the analysed PD-L1$_{IND}$ and PD-L1$_{CON}$ cell lines.

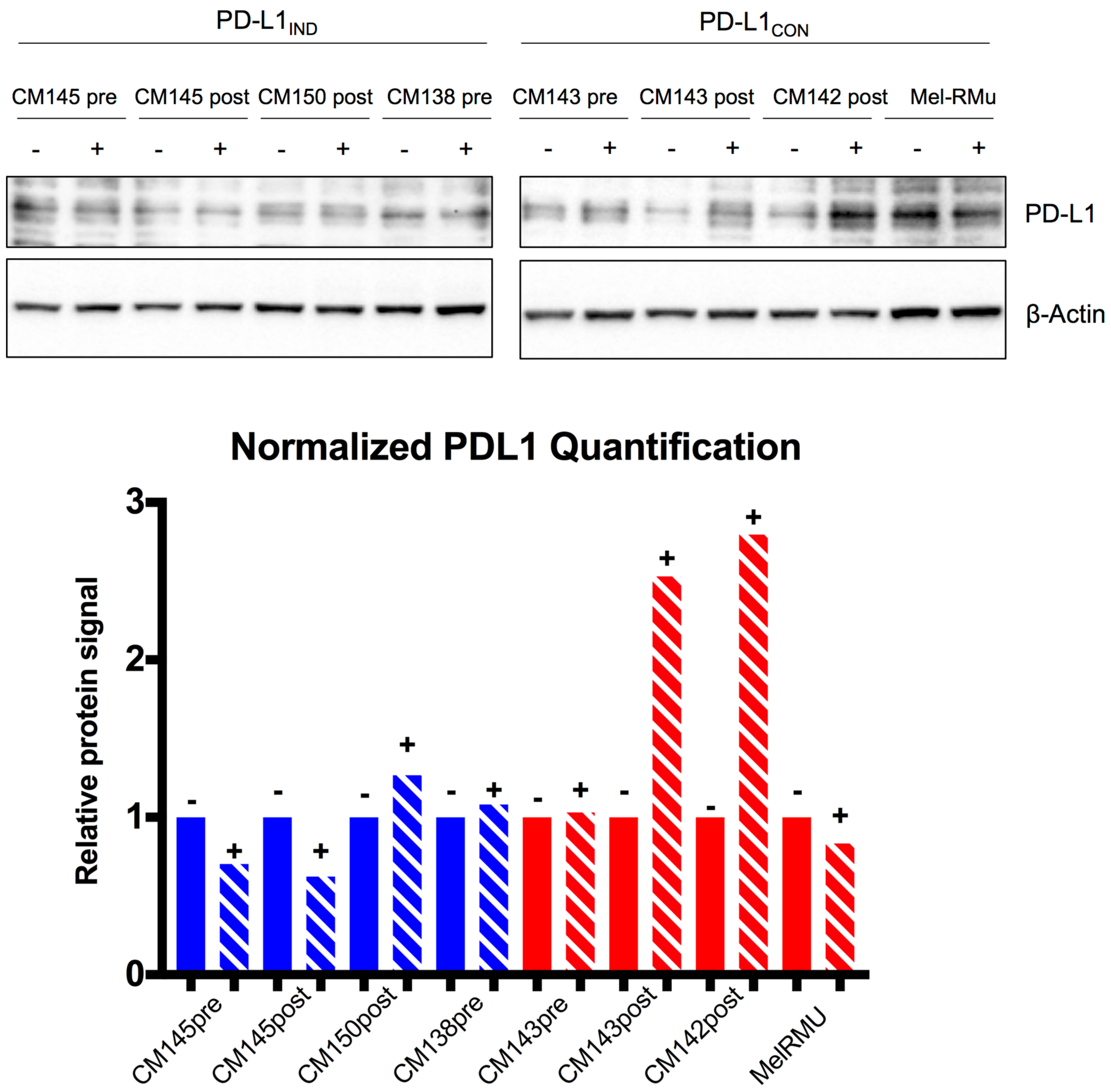

Figure S16. Total protein expression of PD-L1 for PD-L1$_{IND}$ and PD-L1$_{CON}$ groups with vs without decitabine treatment (100nM, 6 days). Related to Figure 6. Western blots of cell lysates were performed with a PD-L1 antibody using a beta-actin antibody as a loading control; representative blots are shown. Protein levels of PD-L1 were normalized against actin and are shown relative to untreated cells. "-" = without decitabine and "+" = with decitabine. All samples were done at least twice. Insufficient samples available for NZM cell lines, therefore these are not shown here.

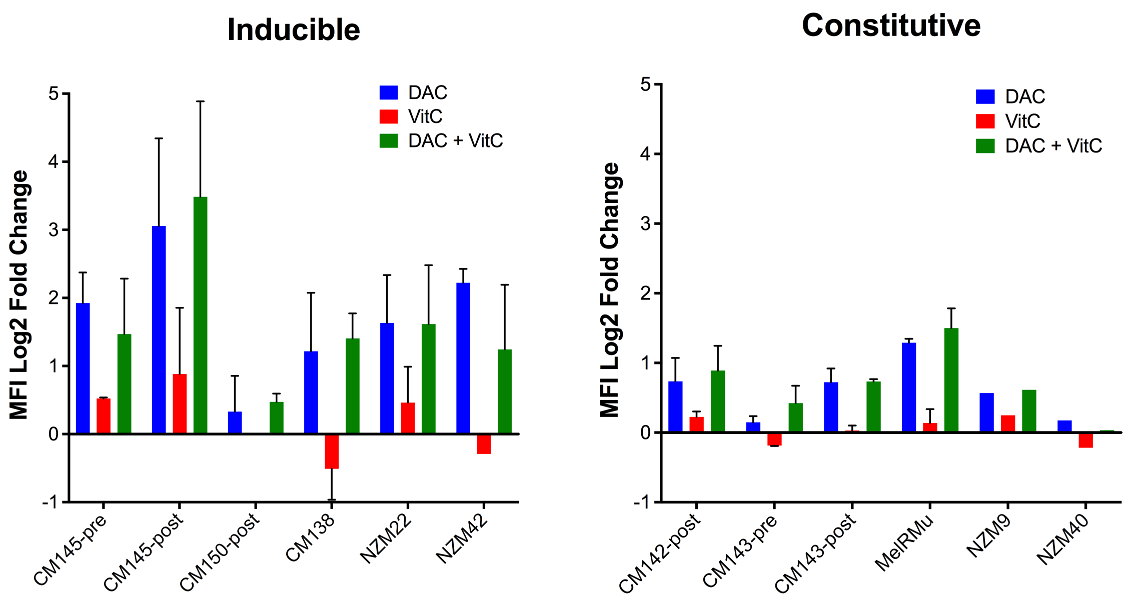

Figure S17. Flow cytometry analysis of PD-L1 cell surface expression upon single and combination treatment of DNMTi (demethylation) and vitamin C in PD-L1$_{IND}$ and PD-L1$_{CON}$ cell lines. Related to Figure 6. Flow cytometry analysis for PD-L1$_{IND}$ (A) and PD-L1$_{CON}$ (B) cell lines were performed at day 6 for all 3 treatment groups of decitabine (DAC), vitamin C (VitC) and decitabine with vitamin C (DAC + VitC). Decitabine (DNMTi; 0.5uM) and mock treatment (DMSO) were performed for 3 daily consecutive days while vitamin C treatment was done for 6 daily consecutive days. PD-L1 expression changes were calculated using medium fluorescence intensities (MFI) using the same formula previously mentioned (see Transparent Methods). Error bars represent SE of two technical replicates.

Supplemental Tables:

Table S1. Percentage of PD-L1 positive cell in the analysed cell lines (from FACS data). Related to Figure 1.

Cell line name	$PD\text{-}L1_{IND}$ or $PD\text{-}L1_{CON}$ Status	% of PD-L1 positive cells	Mutation status
CM145-pre	Inducible	2.22	BRAF V600E
CM145-post	Inducible	1.18	BRAF V600E
CM150-post	Inducible	1.00	BRAF V600E
CM138	Inducible	2.95	BRAF V600E
NZM22	Inducible	6.79	TP53 241S/T/W
NZM42	Inducible	0.82	NRAS Q61K
CM142-post	Constitutive	88.53	BRAF V600E
CM143-pre	Constitutive	83.55	BRAF V600E
CM143-post	Constitutive	98.61	BRAF V600E
MEL-RMU	Constitutive	41.60	BRAF V600E
NZM9	Constitutive	99.07	TP53 179C/T, CDKN2A del
NZM40	Constitutive	98.99	NRAS Q61H, TP53 del249-253, PIK3CA H1047R

Table S2. Details of sequenced reads and mapping for the PD-L1IND and PD-L1CON cell lines. Related to Figure 2.

Cell line name	$PD\text{-}L1_{IND}$ or $PD\text{-}L1_{CON}$ Status	Number of sequenced reads	% of Unique bisulfite mapping
CM145-pre	Inducible	49593852	64.10%
CM145-post	Inducible	17151339	60.90%
CM150-post	Inducible	53768394	64.70%
CM138	Inducible	21470763	61.40%
NZM22	Inducible	42521971	67.10%
NZM42	Inducible	39082074	62.60%
CM142-post	Constitutive	71051964	64.50%
CM143-pre	Constitutive	35395530	67.50%
CM143-post	Constitutive	34604864	67.20%
MEL-RMU	Constitutive	73919898	68.00%
NZM9	Constitutive	43655388	61.80%
NZM40	Constitutive	52776715	64.20%

Table S3. Global methylation profiles in different genomic elements in the PD-L1IND and PD-L1CON cell lines. Related to Figure 2 (see Figure 2A in the main manuscript and the description).

Genome Elements	$PD\text{-}L1_{IND}$		$PD\text{-}L1_{CON}$	
	**Median**	**Mean**	**Median**	**Mean**
Genome-wide	0.63	0.54	0.47	0.48
Promoter	0.06	0.22	0.07	0.22
Intron	0.71	0.57	0.59	0.54
Intron/Exon	0.6	0.5	0.51	0.49
Exon	0.47	0.47	0.43	0.47
Intergenic	0.67	0.59	0.48	0.49

Table S4. Comparison of DNA methylation profiles in different repeat elements for PD-L1IND and PD-L1CON cell lines. Related to Figure 2 (see Figure 2B-2E in the main manuscript and the description).

	Number of Analysed Fragments	$PD\text{-}L1_{IND}$		$PD\text{-}L1_{CON}$	
		**median**	**mean**	**median**	**mean**
**LINEs**					
L1	4851	0.72	0.67	0.53	0.54
L2	4225	0.75	0.67	0.64	0.59
**Satellite elements**					
Telomere	214	0.66	0.6	0.55	0.55
Satellite	305	0.57	0.53	0.39	0.43
Centromere	598	0.58	0.57	0.46	0.48
**SINEs**					
Alu	97416	0.88	0.79	0.79	0.69
MIR	5619	0.71	0.63	0.6	0.56
**LTRs**					
ERV1	11587	0.79	0.72	0.6	0.58
ERVK	875	0.68	0.63	0.52	0.52
ERVL	1984	0.72	0.66	0.57	0.55
ERVL-MalR	2603	0.73	0.68	0.6	0.58

Table S5. Details of DMFs that exhibit a significant positive or negative association of methylation and concomitant differential expression with either PD-L1IND or PD-L1CON cell line groups. Related to Figure 4. Instances where multiple DMFs are associated with a single gene are also shown here.

DMF loci (GRCh37)	PD-L1$_{IND}$ meth	PD-L1$_{CON}$ meth	Diffmeth adjusted P value (FDR 5%)	Overlapping gene	Predicted regulatory feature, target gene(s), Genehancer score	PD-L1$_{IND}$ FPKM	PD-L1$_{CON}$ FPKM	Cuffdiff adjusted P value (FDR 5%)	Spearman correlation of DMF methylation vs target gene[1] expression
chr2:80136765-80136845	0.93	0.23	4.59E-04	*CTNNA2* Exon	-	3.29	0.05	5.35E-03	0.87
chr2:152780685-152780752	0.72	0.13	1.53E-02	*CACNB4* Intron	-	0.06	0.5	1.34E-02	0.07
chr2:171353982-171354051	0.83	0.26	4.07E-02	*MYO3B* Intron	-	0.31	0.01	5.35E-03	0.67
chr3:430086-430153	0.62	0.15	1.61E-03	*CHL1* Intron	-	10.18	0.04	5.35E-03	0.77
chr3:12368678-12368759	0.1	0.89	3.16E-02	*PPARG* Intron	Enhancer: *PPARG* (35.15), *SYN2* (19.35), *TIMP4* (10.18)	0.71	13.97	5.35E-03	0.75
chr3:37540164-37540228	0.86	0.27	2.59E-02	*ITGA9* Intron	-	8.45	0.07	1.09E-02	0.75
chr3:62692712-62692781	0.79	0.23	2.46E-02	*CADPS* Intron	-	4.1	0.23	2.24E-02	0.68
chr3:123120672-123120773	0.74	0.23	3.30E-02	*ADCY5* Intron	-	1.06	0.12	1.34E-02	0.18
chr4:79152150-79152200	0.96	0.24	1.63E-02	*FRAS1* Intron	-	1.74	0.05	5.35E-03	0.82
chr5:155328178-155328245	0.78	0.09	2.74E-02	*SGCD* Intron	-	19.42	0.01	2.38E-02	0.75
chr5:155363892-155363936	0.78	0.12	4.64E-02	*SGCD* Intron	-	19.42	0.01	2.38E-02	0.8
chr5:155404827-155404930	0.81	0.06	6.42E-03	*SGCD* Intron	-	19.42	0.01	2.38E-02	0.77
chr5:155423509-155423595	0.8	0.07	3.10E-02	*SGCD* Intron	-	19.42	0.01	2.38E-02	0.79
chr5:155559547-155559638	0.88	0.12	1.18E-03	*SGCD* Intron	-	19.42	0.01	2.38E-02	0.78
chr5:155675386-155675452	0.89	0.12	3.38E-05	*SGCD* Intron	-	19.42	0.01	2.38E-02	0.8
chr5:155756883-155756950	0.97	0.28	2.20E-02	*SGCD* Intron	Enhancer: *SGCD* (9.64)	19.42	0.01	2.38E-02	0.65
chr5:156028793-156028853	0.96	0.29	7.01E-03	*SGCD* Intron	-	19.42	0.01	2.38E-02	0.89
chr5:156046196-156046328	0.94	0.24	2.11E-03	*SGCD* Intron	-	19.42	0.01	2.38E-02	0.89
chr5:156179564-156179698	0.94	0.23	4.05E-04	*SGCD* Intron	-	19.42	0.01	2.38E-02	0.77
chr5:160111896-160112036	0.83	0.28	3.27E-02	*ATP10B* Intron	Enhancer: *ATP10B* (10.72)	9.32	0.04	5.35E-03	0.7
chr5:160114492-160114627	0.83	0.19	2.20E-02	*ATP10B* Intron	-	9.32	0.04	5.35E-03	0.87
chr5:160201234-160201323	0.9	0.23	8.11E-03	*ATP10B* Intron	-	9.32	0.04	5.35E-03	0.83
chr5:172204336-172204415	0.94	0.31	2.59E-03	Intergenic	Enhancer: *DUSP1* (41.11), *CREBRF* (34.7), *KLF3P1* (20.24), *ERGIC*	10.43	97.48	5.35E-03	-0.65
chr6:401453-401500	0.83	0.42	2.04E-02	*IRF4* Exon	miRNA target site (hsa-miR-205-5p)	12.84	0.08	1.09E-02	0.68
chr7:95583327-95583402	0.86	0.25	1.87E-02	*DYNC1I1* Intron	-	9.84	0.87	1.73E-02	0.21
chr7:151454112-151454226	0.14	0.74	4.55E-02	*PRKAG2* Intron	Enhancer: *PRKAG2* (18.77), *CRYGN* (9.74), *AOC1* (5.42), *SMARCD*	3.54	27.87	5.35E-03	0.68
chr8:95648544-95648611	0.88	0.53	1.69E-02	*ESRP1* Promoter	-	14.51	0.17	5.35E-03	0.73
chr8:120983123-120983191	0.83	0.45	4.23E-02	*DEPTOR* Intron	Enhancer, *COL14A1*, (11.4), *DEPTOR* (25.26), *TAF2* (15.92), *DSCC*	4.07	0.44	2.38E-02	0.29
chr8:121137204-121137324	0.85	0.34	4.87E-02	*COL14A1* Intron	-	4.07	0.44	2.38E-02	0.35
chr8:121206338-121206403	0.83	0.29	3.05E-02	*COL14A1* Intron	-	4.07	0.44	2.38E-02	0.37
chr8:143546627-143546726	0.88	0.32	3.91E-02	*BAI1* Intron	TF binding: *BAI1*	12.44	0.22	5.35E-03	0.8
chr8:143596462-143596531	0.9	0.34	4.91E-02	*BAI1* Intron	-	12.44	0.22	5.35E-03	0.9
chr9:90237682-90237749	0.95	0.45	3.75E-02	*DAPK1* Intron	-	8.03	0.25	5.35E-03	0.71
chr9:90238526-90238632	0.9	0.24	3.06E-03	*DAPK1* Intron	-	8.03	0.25	5.35E-03	0.83
chr9:90292483-90292556	0.85	0.19	2.56E-03	*DAPK1* Intron	-	8.03	0.25	5.35E-03	0.85
chr9:130687304-130687414	0.64	0.91	1.53E-02	*PIP5KL1* Intron/Exon	-	0.48	3.24	5.35E-03	0.76
chr9:130764274-130764342	0.82	0.23	2.59E-03	Intergenic	Enhancer: *PIP5KL1* (15.46), *FAM102A* (12.71), *DPM2* (12.12), *SLC.*	0.48	3.24	5.35E-03	-0.66
chr10:108522243-108522310	0.9	0.18	6.09E-03	*SORCS1* Intron	-	9.27	0.42	2.24E-02	0.74
chr10:108527407-108527543	0.72	0.09	4.65E-02	*SORCS1* Intron	-	9.27	0.42	2.24E-02	0.83
chr10:108554421-108554554	0.77	0.11	2.50E-02	*SORCS1* Intron	-	9.27	0.42	2.24E-02	0.9
chr10:108627203-108627335	0.85	0.16	3.87E-02	*SORCS1* Intron	-	9.27	0.42	2.24E-02	0.81
chr10:108638764-108638831	0.88	0.16	1.77E-02	*SORCS1* Intron	-	9.27	0.42	2.24E-02	0.94
chr10:108689896-108689963	0.75	0.27	4.92E-02	*SORCS1* Intron	-	9.27	0.42	2.24E-02	0.59
chr10:108873305-108873371	0.96	0.25	5.31E-03	*SORCS1* Intron	-	9.27	0.42	2.24E-02	0.68
chr10:124275587-124275651	0.73	0.24	1.76E-02	Intergenic	Enhancer: *DMBT1* (0.24)	0.02	1.14	4.32E-02	-0.69
chr11:17452263-17452344	0.9	0.41	3.26E-02	*ABCC8* Intron	-	0.57	0.05	5.35E-03	0.69
chr11:17796384-17796481	0.55	0.19	4.87E-02	*KCNC1* Intron	-	2.17	0.11	3.80E-02	0.78
chr11:21544483-21544550	0.76	0.05	1.39E-03	*NELL1* Intron	-	1.37	0.04	1.09E-02	0.53
chr11:21544551-21544687	0.71	0.04	2.36E-02	*NELL1* Intron	-	1.37	0.04	1.09E-02	0.81
chr13:24785859-24785904	0.97	0.62	2.21E-03	*SPATA13* Intron	-	12.09	2.91	5.35E-03	0.84

chr13:113734978-113735030	0.9	0.38	4.53E-02	*MCF2L* Intron	-	40.78	4.02	2.10E-02	0.82
chr13:113743774-113743822	0.72	0.34	3.05E-02	*MCF2L* Intron	-	40.78	4.02	2.10E-02	0.8
chr13:114560770-114560828	0.24	0.83	2.53E-02	*GAS6* Intron	Enhancer: *GAS6* (7.24), *GAS6-AS1* (6.28)	8.86	46.94	1.73E-02	0.64
chr14:92336674-92336742	0.86	0.29	2.25E-03	*FBLN5* Intron/Exon	Enhancer, *FBLN5* (16.76), *TC2N* (9.43), *CATSPERB* (8.39)	4.2	1	3.94E-01	0.34
chr15:28098835-28098919	0.84	0.42	2.24E-02	*OCA2* Intron	-	15.47	0.19	4.41E-02	0.5
chr15:28211953-28212063	0.72	0.26	2.22E-02	*OCA2* Intron/Exon	-	15.47	0.19	4.41E-02	0.27
chr15:28249365-28249463	0.62	0.17	2.30E-02	*OCA2* Intron	-	15.47	0.19	4.41E-02	0.27
chr15:28252732-28252799	0.87	0.24	5.31E-03	*OCA2* Intron	-	15.47	0.19	4.41E-02	0.37
chr15:28300866-28300920	0.41	0.05	4.01E-02	*OCA2* Intron	-	15.47	0.19	4.41E-02	0.38
chr15:100694107-100694241	0.92	0.32	4.44E-02	*ADAMTS17* Intron	-	4.2	0.17	5.35E-03	0.79
chr15:100709864-100709979	0.92	0.6	2.90E-02	*ADAMTS17* Intron	-	4.2	0.17	5.35E-03	0.9
chr15:100710120-100710181	0.77	0.13	5.85E-03	*ADAMTS17* Intron	-	4.2	0.17	5.35E-03	0.86
chr15:100773984-100774069	0.96	0.25	1.53E-02	*ADAMTS17* Intron	-	4.2	0.17	5.35E-03	0.81
chr15:101977557-101977611	0.78	0.21	4.40E-02	*PCSK6* Intron	-	6.18	0.67	5.35E-03	0.83
chr16:1029727-1029802	0.87	0.23	4.63E-02	*SOX8* Promoter	-	31.78	0.17	1.09E-02	0.73
chr17:71546113-71546180	0.76	0.22	4.88E-02	*SDK2* Intron	TF binding: *SDK2*	3.19	0.26	5.35E-03	0.79
chr17:71593122-71593178	0.89	0.26	1.01E-02	*SDK2* Intron	-	3.19	0.26	5.35E-03	0.65
chr17:71593179-71593332	0.76	0.24	1.53E-02	*SDK2* Intron	-	3.19	0.26	5.35E-03	0.83
chr17:71607953-71608022	0.87	0.23	4.07E-02	*SDK2* Intron	Enhancer: *SDK2* (18.32)	3.19	0.26	5.35E-03	0.78
chr18:4147291-4147358	0.81	0.12	9.81E-03	*DLGAP1* Intron	-	2.54	0.04	5.35E-03	0.47
chr18:47498335-47498444	0.83	0.25	5.00E-02	*MYO5B* Intron	-	0.35	1.05	6.37E-01	0.21
chr18:74241157-74241268	0.9	0.37	4.92E-02	Intergenic	Enhancer: *ZNF516* (5.24)	7.75	1.9	3.92E-02	0.72
chr19:5909678-5909795	0.25	0.93	1.01E-02	*VMAC* Exon	Enhancer: *CAPS* (33.18), *NDUFA11* (13.52), *VMAC* (0.4)	127.98	2.43	5.35E-03	-0.75
chr19:5910435-5910563	0.1	0.8	2.80E-02	*CAPS* Promoter	-	127.98	2.43	5.35E-03	-0.85

Table S7. Primer Sequences for RT PCR analysis for the 9 HERV genes. Related to Figure 5.

Gene names	Forward sequence	Reverse sequence
MLTA10	TCTCACAATCCTGGAGGCTG	GACCAAGAAGCAAGCCCTCA
MLT1B	TGCCTGTCTCCAAACACAGT	TACGGGCTGAGCTTGAGTTG
MER21C	GGAGCTTCCTGATTGGCAGA	ATGTAGGGTGGCAAGCACTG
ERVL	ATATCCTGCCTGGATGGGGT	GAGCTTCTTAGTCCTCCTGTGT
MLT1C49	TATTGCCGTACTGTGGGCTG	TGGAACAGAGCCCTTCCTTG
MLT1C627	TGTGTCCTCCCCCTTCTCTT	GCCTGTGGATGTGCCCTTAT
MER4D	CCCTAAAGAGGCAGGACACC	TCAAGCAATCGTCAACCAGA
MER57B1	CCTCCTGAGCCAGAGTAGGT	ACCAGTCTGGCTGTTTCTGT
MTL2B4	GGAGAAGCTGATGGTGCAGA	ACCAACCTTCCCAAGCAAGA
SRP14	ACGGAGCTGACCAGACTTTTC	TGGTTCGACCGTCATACTTCTT
RPL27	TGGCTGGAATTGACCGCTAC	CCTTGTGGGCATTAGGTGATTG

Transparent Methods:

Ethics statement: The generation of the cell lines was approved by the Hunter and New England Research Ethics Committee, Australia.

Characterisation of the PD-L1 cell lines: Cell cultures were established as described previously from patients entered into the Roche "BRIM II" phase II study of vemurafenib in patients who had failed previous treatment (Franco et al., 2001). The patient lines were established prior to, and during relapse from treatment with vemurafenib, labelled "pre" and "post" respectively and as described elsewhere (Lai et al., 2012). Cells were cultured in Dulbecco's modified Eagle medium (DMEM) containing 10% fetal calf serum (FCS) (AusGeneX, Brisbane, Australia). All cell lines were tested for mutations using the OncoCARTA and or MelaCARTA panel and contained melanoma associated mutations. In addition, we also used the Geneprint 10 system (Promega, Madison, WI) for authentication and matching of the cell lines.

FACS analysis: The constitutive and inducible status of these cell lines was determined by FACS analysis. The PD-L1 positive percentage was determined using flow cytometry by setting an expression threshold on the isotype control. The gate on the isotype control was set to allow approximately 0.5% of the events to be above the threshold. Subsequently, these gates were applied to the PD-L1 stained samples to determine the PD-L1 positive percentage. All the analysed melanoma cell lines were stained with anti-PD-L1 (PE) and the isotype control antibody. An expression level of at least fivefold higher than the isotype control was considered as "constitutive" expression of PD-L1 (see FACS analysis figures in Supplemental Figure S1-S2).

Interferon blocking experiment: PD-L1$_{CON}$ cell lines CM143-pre and CM143-post were treated with blocking antibodies against interferon a receptor 2 (IFNAR2) (clone MMHAR-2, PBL Assay Science, Piscataway, NJ, 10mg/ml), IFNgR1 (clone GIR20s8, R&D Systems, Minneapolis, MN, 5mg/ml), or IFNg (clone NIB42, Beckton Dickinson, Franklin Lakes, NJ, 100ng/ml) in the absence or presence of 10^4 U IFNa2A (Stemcell Technologies, Vancouver, Canada) or 100ng/ml IFN-g (R&D Systems, Minneapolis, MN), as appropriate. PD-L1 expression was measured by flow cytometry on day 3.

RRBS library preparation and sequencing: We used reduced representation bisulfite sequencing (RRBS) to map promoter and gene body DNA methylation as described previously (Chatterjee et al., 2016a; Chatterjee et al., 2013; Chatterjee et al., 2012a; Chatterjee et al., 2014; Chatterjee et al., 2015b). Briefly, genomic DNA was digested with MspI followed by end-repair and ligation of sequencing adaptors. The fragments were size selected and bisulfite-converted prior to a PCR amplification step. The quality and size distribution of the libraries was determined using a bioanalyser and four libraries were sequenced per flow cell lane of an Illumina HiSeq2500 machine (100 bp reads, single-ended).

DNA methylation data analysis: The quality check and processing of the sequenced RRBS reads was performed using in-house developed bioinformatics tools as previously described (Chatterjee et al., 2012b; Stockwell et al., 2014). The Bismark tool (Krueger and Andrews, 2011) was used to align the processed sequence reads to the reference human genome (GRCh37). We applied stringent mapping criteria by

allowing only one mismatch (default = 2) in the seed (i.e., in the first 28 bp of the sequenced reads). After filtering for low quality sequences, we obtained > 60% unique alignment for all the PD-L1$_{CON}$ and PD-L1$_{IND}$ RRBS libraries, respectively. The median non-CpG DNA methylation was 1.95% and 2.45% in the PD-L1$_{CON}$ and PD-L1$_{IND}$ libraries, respectively (as measured by Bismark alignment), indicating effective bisulfite conversion and low levels of true non-CpG methylation.

The distribution and level of CpG DNA methylation (on a scale of 0–1) was determined, using MspI fragments (40–220 bp) as the unit of analysis rather than individual CpG sites or a tiled window approach, as previously described (Chatterjee et al., 2016b; Chatterjee et al., 2017; Chatterjee et al., 2015b; Chatterjee et al., 2016c). Differential methylation analysis was performed with an in-house Differential Methylation Analysis Pipeline (DMAP), which contains two main programmes (diffmeth and identgenloc) (Stockwell et al., 2014). Briefly, we applied an F statistic (ANOVA test) on fragments that had high quality methylation information (at least two CpG sites covered by 10 or more sequenced reads, -F 2 -t 10 switch in the diffmeth program of the DMAP tool) in at least 3 cell lines in each group, and identified regions showing the largest methylation difference and significant *P*-values. We applied a false discovery rate of 5% on the analysed fragments (at an alpha level = 0.05) to filter for significant fragments. We further filtered this list and obtained fragments with 0.25 (i.e., 25%) of higher methylation difference (mean methylation on fragments) between the PD-L1$_{CON}$ and PD-L1$_{IND}$ groups.

RNA isolation and construction of RNA-Seq libraries: RNA was extracted from cell lines using an RNeasy Plus mini prep kit (QIAGEN, Limburg, Netherlands), and quantified using Nanodrop (Thermo Scientific, Wilmington, DE) as previously described (Chatterjee et al., 2015a; Leichter et al., 2015). RNA quality was assessed using Bioanalyser analysis of RNA integrity number (RIN) (Agilent, USA). RNA libraries were constructed using 1μg of total RNA with a TruSeq stranded mRNA Sample Preparation kit (Illumina) following the manufacturer's protocol. Briefly, poly-A containing mRNA was purified using oligo-dT magnetic beads. Next, RNA fragments were reverse transcribed using random primers and reverse transcriptase and first strand cDNA was synthesized. Following this, second strand cDNA was synthesized, and the cDNA was blunt-ended, which was followed by 'A' tailing and adaptor ligation. The adaptor ligated cDNA was amplified by PCR for sequencing.

Analysis of transcriptomic data: RNA was sequenced on an Illumina HiSeq 2500 sequencer (Illumina, USA) with paired-end, 101-bp runs producing raw fastq files. The RNA-seq reads were adaptor trimmed using the cleanadaptors tool (Chatterjee et al., 2012b) and mapped to the human genome (assembly GRCh37) using TopHat2 (Kim et al., 2013). Transcripts were assembled and normalized gene expression levels were expressed in FPKM (Fragments Per Kilo base per Million) values as generated by cuffquant and cuffnorm programmes (Trapnell et al., 2012). Assembly of transcripts and generation of the FPKM values was performed with the option ''–frag-bias- correct'' and ''–multi-read-correct'' to improve sensitivity of transcript detection (Roberts et al., 2011). We identified 557 genes that were significantly differentially expressed (DEG) between PD-L1$_{IND}$ and PD-L1$_{CON}$ cell lines (P-value <0.05, FDR corrected). We further filtered this list based on fold-expression change and selected the genes that showed $\log_2$ fold change of mean FPKM (fragment per kilobase per million read) ≥ 2, resulting in 508 DEGs (analysed with cuffdiff

(Trapnell et al., 2012)). The list of epigenetic regulator genes was obtained from EpiFactors database (Medvedeva et al., 2015). Pathway analysis on DEGs was performed using Metascape (METASCAPE.ORG).

TCGA data analysis and deconvolution of based on TIL and PD-L1 expression: TCGA Firehose level 3 data for skin cutaneous melanoma was downloaded using an R package (Samur, 2014). Clinical details, methylation 450k and RNAseqV2 with run date "20151101" were selected for download. The RNA-Seq data are RSEM normalised values with expected counts for each gene. Samples were filtered to 469 samples after retaining only those samples with information on all three of the following; clinical details, methylation 450k and RNA-seq data. RNA-seq data was used to generate three values that infered the quantity of CD8 TILs using computational tools including CiberSort (Newman et al., 2015) MCPcounter (Becht et al., 2016) and xCell (Aran et al., 2017). Methylation 450k data were used to generate the meTIL (methylation TIL) score. This was calculated with beta-values from five CpG probes using the formula provided by Jeschke et al (Jeschke et al., 2017). To obtain the average TIL score from these four variables, first all zero CD8 values produced from CiberSort, MCPcounter and xCell were converted to half of the smallest value in that corresponding variable. These three variables were logarithmically transformed. All four variables were scaled to generate Z-scores by subtracting the mean and dividing by the standard deviation. The average TIL score was then calculated for each sample using the arithmetic mean. The average TIL-score and PD-L1 mRNA values were used to generate four groups from 469 samples according to high and low presence of TILs and PD-L1 expression. First, samples were split according to high and low TIL-score values using the median as a cut-off threshold. Samples were then split again using the median PD-L1 mRNA expression levels as the cut-off. Groups 1 and 2 represent the inducible and constitutive patient groups (see Supplemental Figure S9-S10 for their TIL and PD-L1 levels).

qPCR analysis of HERV genes: Total RNA was extracted using the RNeasy Mini Kit (Qiagen) and reverse transcription was performed using the High-Capacity cDNA Reverse Transcription Kit (ThermoFisher). RT-PCR was run on the Light Cycler 480 (Roche) using SYBR green (SYBR Premix Ex Taq II, Takara). Primers were acquired from Roulois et al (Roulois 2015) and are listed in Supplemental Table S7. Gene expressions were normalised to house-keeping genes RPL27 and SPR14 and analysis was done using the qbaseplus software (Biogazelle).

Western blot analysis: Cell pellets were washed in ice-cold PBS and lysed using RIPA buffer containing a protease inhibitor cocktail. After centrifugation, supernatants were collected and total protein quantified using the Biorad DC protein assay. 20-40µg of total protein was electrophoresed on 8-12% SDS-PAGE gels and transferred onto nitrocellulose membranes. Membranes were blocked and incubated overnight in one of the following primary antibodies: PD-L1 (#AF156, R&D Systems), DNMT3A (#3598, Cell Signalling), UHRF1 (#12387, Cell Signalling), UHRF2 (#PA5-40969, Thermo Fisher), ß-actin (#A2228, Sigma) and α-tubulin (#T5168, Sigma). Following incubation in the appropriate HRP-conjugated secondary antibodies, chemiluminescent imaging was performed on a Chemidoc imaging system. Western blot images were analysed using LI-COR Image Studio software.

Data availability: DNA methylation and transcriptomic data for PD-$L1_{CON}$ and PD-$L1_{IND}$ cell lines are available at Database: NCBI GEO, accession number GSE107622.

DNMTi treatment of PD-L1 cell lines and data analysis: All twelve cell lines were treated with decitabine (500nM; Sigma Aldrich, DNMTi treatment) or DMSO (Sigma Aldrich), while changing the media containing fresh drug or vehicle daily, for three consecutive days. Subsequently, cells were grown in fresh media (without drug) for an additional three days. At day six cells were harvested for flow cytometry analysis. An independent biological replicate study was carried out using the same experimental conditions, and treated with 100nM decitabine, generated essentially the same results. Western blots were performed to confirm PD-L1 protein levels upon demethylation (Supplemental Figure S11). Flow cytometry analysis was performed as described above. Changes of PD-L1 expression between decitabine-treated and control were calculated using medium fluorescence intensities (MFI) and the formula: $\log_2([(MFI_{antibody,\ treated})-(MFI_{isotype,\ treated})]/\ [(MFI_{antibody,\ mock})-(MFI_{isotype,\ mock})])$ (Wrangle et al., 2013).

Supplemental References:

Aran, D., Hu, Z., and Butte, A.J. (2017). xCell: digitally portraying the tissue cellular heterogeneity landscape. Genome Biol *18*, 220.

Becht, E., Giraldo, N.A., Lacroix, L., Buttard, B., Elarouci, N., Petitprez, F., Selves, J., Laurent-Puig, P., Sautes-Fridman, C., Fridman, W.H.*, et al.* (2016). Estimating the population abundance of tissue-infiltrating immune and stromal cell populations using gene expression. Genome Biol *17*, 218.

Chatterjee, A., Lagisz, M., Rodger, E.J., Zhen, L., Stockwell, P.A., Duncan, E.J., Horsfield, J.A., Jeyakani, J., Mathavan, S., Ozaki, Y.*, et al.* (2016a). Sex differences in DNA methylation and expression in zebrafish brain: a test of an extended 'male sex drive' hypothesis. Gene *590*, 307-316.

Chatterjee, A., Leichter, A.L., Fan, V., Tsai, P., Purcell, R.V., Sullivan, M.J., and Eccles, M.R. (2015a). A cross comparison of technologies for the detection of microRNAs in clinical FFPE samples of hepatoblastoma patients. Sci Rep *5*, 10438.

Chatterjee, A., Macaulay, E.C., Rodger, E.J., Stockwell, P.A., Parry, M.F., Roberts, H.E., Slatter, T.L., Hung, N.A., Devenish, C.J., and Morison, I.M. (2016b). Placental Hypomethylation Is More Pronounced in Genomic Loci Devoid of Retroelements. G3 (Bethesda) *6*, 1911-1921.

Chatterjee, A., Ozaki, Y., Stockwell, P.A., Horsfield, J.A., Morison, I.M., and Nakagawa, S. (2013). Mapping the zebrafish brain methylome using reduced representation bisulfite sequencing. Epigenetics *8*, 979-989.

Chatterjee, A., Rodger, E.J., Stockwell, P.A., Weeks, R.J., and Morison, I.M. (2012a). Technical considerations for reduced representation bisulfite sequencing with multiplexed libraries. J Biomed Biotechnol *2012*, 741542.

Chatterjee, A., Stockwell, P.A., Ahn, A., Rodger, E.J., Leichter, A.L., and Eccles, M.R. (2017). Genome-wide methylation sequencing of paired primary and metastatic cell lines identifies common DNA methylation changes and a role for EBF3 as a candidate epigenetic driver of melanoma metastasis. Oncotarget *8*, 6085-6101.

Chatterjee, A., Stockwell, P.A., Horsfield, J.A., Morison, I.M., and Nakagawa, S. (2014). Base-resolution DNA methylation landscape of zebrafish brain and liver. Genom Data *2*, 342-344.

Chatterjee, A., Stockwell, P.A., Rodger, E.J., Duncan, E.J., Parry, M.F., Weeks, R.J., and Morison, I.M. (2015b). Genome-wide DNA methylation map of human

neutrophils reveals widespread inter-individual epigenetic variation. Sci Rep *5*, 17328.
Chatterjee, A., Stockwell, P.A., Rodger, E.J., and Morison, I.M. (2012b). Comparison of alignment software for genome-wide bisulphite sequence data. Nucleic Acids Res *40*, e79.
Chatterjee, A., Stockwell, P.A., Rodger, E.J., and Morison, I.M. (2016c). Genome-scale DNA methylome and transcriptome profiling of human neutrophils. Sci Data *3*, 160019.
Franco, A.V., Zhang, X.D., Van Berkel, E., Sanders, J.E., Zhang, X.Y., Thomas, W.D., Nguyen, T., and Hersey, P. (2001). The role of NF-kappa B in TNF-related apoptosis-inducing ligand (TRAIL)-induced apoptosis of melanoma cells. J Immunol *166*, 5337-5345.
Jeschke, J., Bizet, M., Desmedt, C., Calonne, E., Dedeurwaerder, S., Garaud, S., Koch, A., Larsimont, D., Salgado, R., Van den Eynden, G., *et al.* (2017). DNA methylation-based immune response signature improves patient diagnosis in multiple cancers. J Clin Invest *127*, 3090-3102.
Kim, D., Pertea, G., Trapnell, C., Pimentel, H., Kelley, R., and Salzberg, S.L. (2013). TopHat2: accurate alignment of transcriptomes in the presence of insertions, deletions and gene fusions. Genome Biol *14*, R36.
Krueger, F., and Andrews, S.R. (2011). Bismark: a flexible aligner and methylation caller for Bisulfite-Seq applications. Bioinformatics *27*, 1571-1572.
Lai, F., Jiang, C.C., Farrelly, M.L., Zhang, X.D., and Hersey, P. (2012). Evidence for upregulation of Bim and the splicing factor SRp55 in melanoma cells from patients treated with selective BRAF inhibitors. Melanoma Res *22*, 244-251.
Leichter, A.L., Purcell, R.V., Sullivan, M.J., Eccles, M.R., and Chatterjee, A. (2015). Multi-platform microRNA profiling of hepatoblastoma patients using formalin fixed paraffin embedded archival samples. Gigascience *4*, 54.
Medvedeva, Y.A., Lennartsson, A., Ehsani, R., Kulakovskiy, I.V., Vorontsov, I.E., Panahandeh, P., Khimulya, G., Kasukawa, T., Consortium, F., and Drablos, F. (2015). EpiFactors: a comprehensive database of human epigenetic factors and complexes. Database (Oxford) *2015*, bav067.
Newman, A.M., Liu, C.L., Green, M.R., Gentles, A.J., Feng, W., Xu, Y., Hoang, C.D., Diehn, M., and Alizadeh, A.A. (2015). Robust enumeration of cell subsets from tissue expression profiles. Nat Methods *12*, 453-457.
Roberts, A., Trapnell, C., Donaghey, J., Rinn, J.L., and Pachter, L. (2011). Improving RNA-Seq expression estimates by correcting for fragment bias. Genome Biol *12*, R22.
Samur, M.K. (2014). RTCGAToolbox: a new tool for exporting TCGA Firehose data. PLoS One *9*, e106397.
Stockwell, P.A., Chatterjee, A., Rodger, E.J., and Morison, I.M. (2014). DMAP: differential methylation analysis package for RRBS and WGBS data. Bioinformatics *30*, 1814-1822.
Trapnell, C., Roberts, A., Goff, L., Pertea, G., Kim, D., Kelley, D.R., Pimentel, H., Salzberg, S.L., Rinn, J.L., and Pachter, L. (2012). Differential gene and transcript expression analysis of RNA-seq experiments with TopHat and Cufflinks. Nat Protoc *7*, 562-578.
